# Dysregulated ac4C modification of mRNA in a mouse model of early-stage Alzheimer’s disease

**DOI:** 10.1186/s13578-025-01389-8

**Published:** 2025-04-13

**Authors:** Hao-Nan Ji, Hai-Qian Zhou, Jing-Bo Qie, Wen-Mei Lu, Hai-Tao Gao, Dan-Hong Wu

**Affiliations:** 1 Department of Neurology, Shanghai Fifth People’s Hospital, Fudan University, Shanghai, 200240 China; 2 Shanghai Fifth People’s Hospital, Institutes of Biomedical Sciences, Fudan University, Shanghai, 200032 China; 3 Center of Community-Based Health Research, Fudan University, Shanghai, 200032 China

**Keywords:** ac4C, Gene transcription, Proteomics, Alzheimer’s disease, Hippocampus

## Abstract

**Background:**

The identification and intervention of Alzheimer’s Disease (AD) in its early-stage allows for the timely implementation of lifestyle modifications and therapeutic strategies. Although dysregulation of protein expression has been reported in the brain from AD patients and AD animal models, the underlying mechanisms remain poorly understood. N4-acetylcytidine (ac4C), the only known form of RNA acetylation in eukaryotes, has recently been shown to regulate mRNA stability and translation efficiency. However, the dysregulation of ac4C associated with abnormal protein expression levels in the brain of early-stage mouse models of AD remains to be elucidated.

**Methods:**

This study investigated ac4C modifications, mRNA and protein expression in the hippocampus of 3 and 6-month-old 5×FAD mice, a mouse model of AD, and wild-type (WT) littermates. The multi-omics analysis was performed: acetylated RNA immunoprecipitation followed by next-generation sequencing (acRIP-seq) to identify ac4C mRNAs, deep RNA sequencing (RNA-seq) to quantify mRNA abundance, and label-free quantitative proteomics to assess protein expression levels. In addition, we used acRIP-qPCR, regular qPCR and western blots to verify the ac4C, mRNA and protein levels of some key genes that were identified by the high-throughput assays.

**Results:**

Proteomic analysis revealed significant change of protein expression in the hippocampus of 3-months-old 5×FAD mice, compared with WT littermates. In contrast, RNA-seq analysis indicated that there were no substantial alterations in mRNA expression levels in the hippocampus of 3-months-old 5×FAD mice, compared to WT littermates. Strikingly, acRIP-seq revealed notable variations in ac4C modification on mRNAs, particularly those associated with synaptic structure and function, in the hippocampus of 3-months-old 5×FAD mice, compared with WT littermates. The ac4C modifications were found to be correlated with protein expression changes. Genes that are essential for synaptic function and cognition, including GRIN1, MAP2, and DNAJC6, exhibited reduced ac4C and protein levels in 3-months-old 5×FAD mice, without any corresponding changes in the mRNA levels, compared with WT littermates. Moreover, only a small part of dysregulated ac4C mRNAs identified in the 3-month-old 5×FAD mice were found in the 6-month-old 5×FAD mice.

**Conclusions:**

Altogether these results identified abnormal ac4C modification of mRNAs that may contribute to the dysregulation of protein synthesis in the hippocampus from an early-stage mouse model of AD.

**Supplementary Information:**

The online version contains supplementary material available at 10.1186/s13578-025-01389-8.

## Background

Alzheimer’s disease (AD) is a complex neurodegenerative disorder characterized by progressive cognitive decline and memory loss [1]. AD is a prevalent central nervous system disorder that can lead to neuropathological alterations in specific brain regions, including the hippocampus, temporal cortex, and amygdala [2]. As the most common form of dementia, AD affects millions worldwide and places a significant burden on healthcare systems. The pathological hallmarks of AD include the accumulation of amyloid-beta (Aβ) plaques, hyperphosphorylated tau tangles, neuroinflammation, and neuronal loss [3]. Although extensive research has focused on these hallmark features, the early molecular mechanisms driving AD pathology remain inadequately understood. Furthermore, current therapeutic options for AD are limited, with irreversible neuronal damage posing a significant obstacle to effective treatment. Understanding the pathological alterations occurring in the early-stages of AD—when cognitive function is largely preserved—is particularly important [4, 5]. Intervention during early-stages could prevent or delay cognitive decline, providing a critical window of opportunity to mitigate disease progression before irreversible damage occurs. Therefore, identifying these early molecular mechanisms is essential for developing preventive therapies that preserve cognitive function and improve patient outcomes. Discovering these initial pathways is crucial for advancing preventive strategies and novel therapeutic targets that delay or halt AD progression.

One critical aspect of gene regulation that has gained attention in recent years is post-transcriptional modifications, whichsignificantly modulate RNA stability and translation efficiency. Post-transcriptional modifications of mRNA, including N6-methyladenosine (m6A), N4-acetylcytidine (ac4C), and 5-methylcytosine (m5C), are essential for the proper functioning of mRNAs. These modifications can influence mRNA metabolism by altering splicing, export from the nucleus, and translation efficiency [6]. For example, m6A modification, extensively studied for its role in regulating RNA stability and translation across various cellular contexts, has been the primary focus of epitranscriptomic research in neurodegenerative diseases [7, 8]. In contrast, the roles of RNA acetylation in the pathogenesis neurodegenerative diseases are relatively unexplored. Ac4C is the only known form of RNA acetylation in eukaryotes [9]. Studies in cell biology have elucidated the mechanisms by which ac4C modifications influence mRNA stability and translation efficiency [10, 11]. Ac4C modifications significantly enhance mRNA stability through increasing the interaction of mRNA with the ac4C writer NAT10. On the other hand, mRNA acetylation reciprocally enhances translation by promoting interaction with cognate tRNAs. Our recent study indicated that the stoichiometry levels of ac4C were 2-fold higher than m6A in mouse hippocampus [12], which suggest that ac4C may be a previously-ignored epitranscriptomic mechanism regulating hippocampal functions. In addition, NAT10, the only known ac4C writer has been implicated in aging-related diseases [13, 14]. Furthermore, the dysregulation of energy metabolism is a hallmark of AD, and acetyl-CoA, a central metabolite in cellular energy pathways, plays a critical role in this process [15, 16]. It is notable that ac4C modifications requires acetyl groups derived from acetyl-CoA, and thus fluctuations in acetyl-CoA levels in AD may directly impact ac4C deposition. Recent studies have begun to elucidate the role of NAT10 (N-acetyltransferase 10)-mediated ac4C modifications in various physiological and pathological processes [17], particularly in neurodegenerative diseases [18]. These functions underscore the potential of ac4C modifications as therapeutic targets in neurodegenerative diseases like AD, where cellular stability, protein homeostasis, and neuronal health are critically compromised. Our previous research has also revealed that neural activities, such as learning and memory, can specifically regulate ac4C-mRNA modification levels at synaptic sites. The ac4C-mRNA at synapses is upregulated following memory formation but returns to baseline levels after natural forgetting, demonstrating that the regulation of ac4C by learning and memory is localized, dynamic, and specific [12]. Given the role of ac4C in regulating mRNA dynamics [12, 19], alterations in ac4C modification could contribute to the molecular pathophysiology of AD. However, the results from previous studies did not reveal the transcriptional regulatory mechanisms at the early-stages of AD.

Recent advances in sequencing technologies have greatly enhanced our understanding of gene expression and protein dynamics at the molecular level. In this context, we have used protein sequencing to analyze expression profiles and post-translational modifications critical for elucidating disease mechanisms. Similarly, we have used mRNA sequencing to delineate the transcriptomic landscape, capturing a comprehensive set of expression patterns and splicing variants critical during key stages of cellular development or disease progression. we used 5×FAD mice in this study to facilitate targeted studies of early-stage Alzheimer’s disease. The 3-month-old 5×FAD mice have been widely used as an early-stage mouse model of AD due to the following reasons. It exhibits early Aβ plaque formation that align with preclinical stages of AD in humans [20, 21]. However, no neurite dystrophy, neuronal loss or obvious cognitive deficit were observed at 3 months old in 5×FAD mice [22]. In contrast, deficient synaptic plasticity such as impaired LTP, and neuroinflammation such as astrogliosis and microgliosis may appear at 3 months old in 5×FAD mice [23–25]. Cognitive deficits like impaired spatial and working memory usually start from 5 to 6 months old in 5×FAD mice [26]. Progressive neuronal loss usually begins at 6-months old in brain regions with pronounced amyloidosis [27]. We constructed a detailed map of gene and protein expression using these advanced molecular techniques. In addition, we used acetylation RNA immunoprecipitation sequencing (acRIP-seq) to study changes in acetylated RNA in the early-stages of AD. This approach is critical for elucidating regulatory mechanisms at the RNA level, particularly post-transcriptional modifications that affect RNA stability and translation efficiency. By integrating data from protein sequencing, mRNA sequencing, and acRIP-seq, our goal is to provide a comprehensive view of the molecular dynamics involved, thereby providing deeper insights into cellular functions and the pathological basis of AD. Moreover, orthogonal experiments confirmed the significance of key early AD genes at the protein, mRNA and ac4C-modified mRNA levels. This comprehensive validation approach serves to reinforce the reliability of sequencing results, emphasize the pivotal role of these biomarkers in the pathogenesis of early AD and establish a robust framework for understanding post-transcriptional functions in early AD.

## Results

### Amyloid pathology manifests in 3-month-old 5×FAD mice without concomitant cognitive deficits

**Fig. 1:**
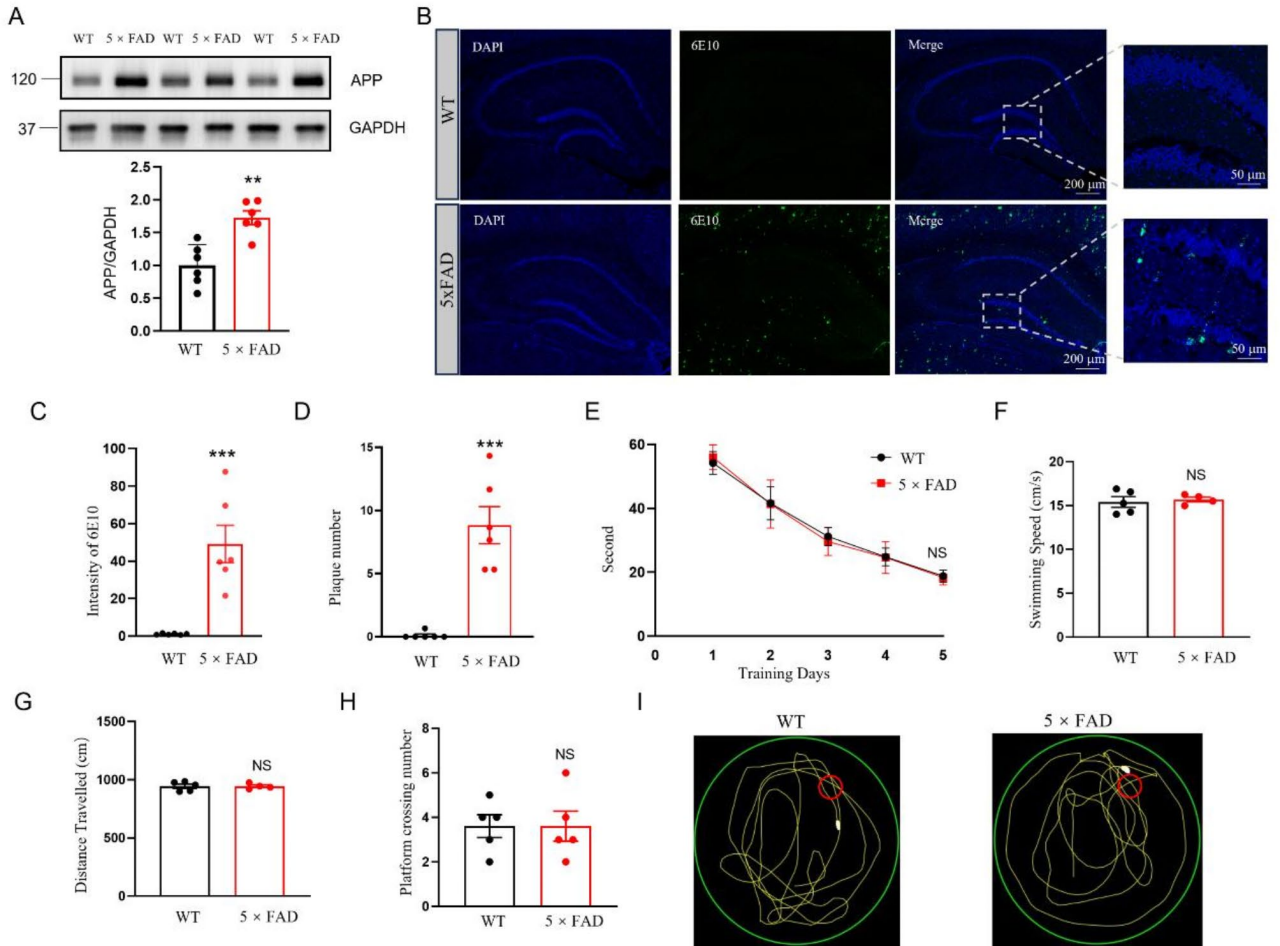
Molecular and behavioral validation of early Alzheimer’s disease pathology in 3-month-old 5×FAD mice. **A** Western Blot Analysis: Shows the protein expression levels of APP in the hippocampus. The higher expression levels in 3-month-old 5×FAD mice compared to WT controls demonstrate the successful expression of Alzheimer’s disease-related transgenes. Top, representative western blot images; bottom, quantification data. *n* = 6 biological replicates at each condition, ** *P* = 0.0013, two-tailed t-test. Data were normalized by WT. **B-D** Immunohistochemistry for Amyloid Beta (6E10): **B** Overview showing amyloid plaque distribution in the hippocampus. Scale bars: left, 200 μm and right, 50 μm. **C** Quantification of amyloid beta intensity in 5×FAD mice and WT mice. **D** Quantification of amyloid beta plaque accumulation, substantiating increased plaque number in 5×FAD mice versus WT controls. **E** In the Morris water maze test, there were no significant differences in cognitive performance between 5×FAD mice and WT mice, suggesting the absence of early cognitive decline in this model at 3 months of age. *n* = 5 mice, NS, not significant, one-way ANOVA. **F** Compares the swimming speed between 5×FAD and WT mice, showing no impairments in motor functions: NS, not significant, two-tailed t-test. **G** Analysis showing the distance traveled during the Morris water maze test, with similar results between 5×FAD and WT mice, indicating normal locomotor activity. NS, not significant, two-tailed t-test. **H** Total number of platform crossings. NS, not significant, two-tailed t-test. **I** Detailed tracked paths from the Morris water maze, demonstrating no significant differences in memory recall or learning ability between the two groups

We used 5×FAD transgenic mice, a widely used model, to overexpress human amyloid precursor protein (APP695) with the Swedish (K670N/M671L), Florida (I716V), and London (V717I) mutations under the control of the murine Thy-1 promoter, and human presenilin-1 (PS1) with the M146L and L286V mutations [28]. The model was established using C57BL/6J background embryos via standard pronuclear injection techniques. Western blot analysis of hippocampal protein extracts from 3-month-old 5×FAD mice confirmed increased APP expression compared to WT controls (Fig. 1A), consistent with previous studies [23, 29]. Anti-Aβ1–16 (6E10) is a widely used monoclonal antibody that specifically recognizes APP and its cleavage product, Aβ, specifically Aβ (1–16/17) [30]. To assess the accumulation of amyloid plaques, we performed immunohistochemistry using the 6E10 on hippocampal sections from 3-month-old 5×FAD mice. Immunohistochemistry using the 6E10 revealed significant amyloid plaque accumulation in 3-month-old 5×FAD mice (Fig. 1B, C, D). Behavioral tests, including the Morris water maze, showed no significant differences in cognitive performance, swimming speed, or distance traveled between 5×FAD and WT mice (Fig. 1E-H). These findings align with numerous studies [21, 28, 31], which also indicate that while 5×FAD mice show early pathological characteristics, such as increased APP expression and β-amyloid plaques, cognitive impairments are not observed at 3 months of age.

### Comparative proteomic profiling of hippocampal tissue from 3-month-old WT and 5×FAD mice

**Fig. 2:**
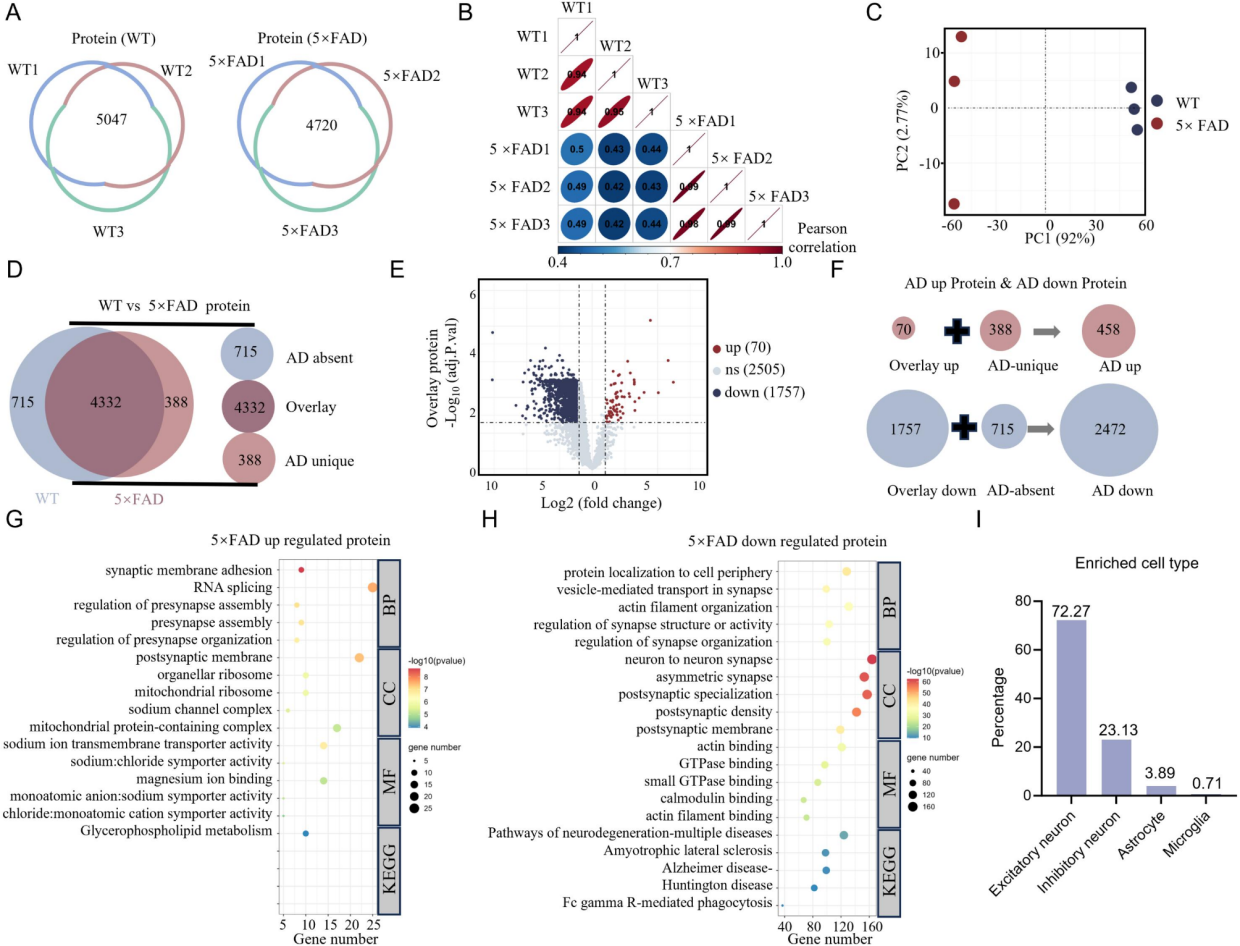
Proteomic analysis of the hippocampus of 3-month-old WT and 5×FAD mice. **A** Proteomics-based LC-MS/MS analysis was performed on the hippocampus of 3-month-old WT and 5×FAD mice in three independent biological replicates, identifying 5904 and 4838 proteins, and successfully quantifying 5047 and 4720 proteins in WT and 5×FAD mice after MBR, respectively. **B** Correlation analysis revealed higher intra-group correlations than inter-group correlations. All samples showed higher intra-group correlations, with the correlation values visualized in an ellipse plot. The shape of the ellipse corresponds to the absolute value of the correlation, with flatter ellipses indicating stronger correlations. Black numbers represent pairwise Spearman’s correlation coefficients between the indicated samples. **C** The top 50% of proteins, sorted by mean absolute deviation (MAD), were selected for principal component analysis (PCA). The PCA plot showed a clear clustering of the samples, with blue dots representing WT mice and red dots representing 5×FAD mice. **D** A Venn diagram illustrating the overlap of proteins detected in both WT and 5×FAD mice. “AD absent” refers to proteins detected only in WT mice, “Overlay” refers to proteins detected in both groups, and “AD unique” refers to proteins detected only in 5×FAD mice. **E** Volcano plot of overlay proteins showed that 1757 proteins were downregulated in the 5×FAD group compared to WT, while only 70 proteins were upregulated. Differentially expressed proteins were defined as having an adjusted p-value < 0.05 and a fold change > 2, with statistical correction using the least squares method and Bayesian adjustments. Blue dots indicate downregulated proteins, red dots represent upregulated proteins, and gray dots are non-significantly different proteins. **F** The composition of differentially expressed proteins is detailed, with 458 upregulated proteins in 5×FAD mice. These include the 70 overlay upregulated proteins and 388 AD unique proteins. In addition, 2472 downregulated proteins were identified, consisting of 1757 overlay downregulated proteins and 715 AD-unique proteins. **G**-**H** GO and KEGG pathway enrichment analyses were performed for the differentially expressed proteins. For the upregulated proteins, the top 5 Biological Processes (BP), Molecular Functions (MF), Cellular Components (CC), and KEGG pathways were summarized (G). Similar analyses were performed for the downregulated proteins (H), with an adjusted p-value threshold of < 0.05. **I** The bar graph show enriched cell types for the expression of decreased proteins. 72.27% of these proteins were predominantly expressed in excitatory neurons and 23.13% were enriched in GABAergic interneurons, while a smaller proportion (4.6%) were enriched in glial cells

To investigate proteomic changes in the hippocampus associated with early AD, we performed a comparative proteome analysis of the hippocampus of 3-month-old WT and 5×FAD mice. A total of 5904 proteins were identified in the WT group and 4838 in the 5×FAD group, of which 5047 and 4720 proteins were successfully quantified after match between run (MBR) correction (Fig. 2A). Correlation analysis showed higher intra-group consistency compared to inter-group samples (Fig. 2B), and Principal Component Analysis (PCA) revealed distinct clustering between WT and 5×FAD samples, highlighting divergent proteiome profiles (Fig. 2C). A Venn diagram highlighting distinct protein subsets, with 715 proteins uniquely present in WT mice (“AD absent”), 388 proteins specific to 5×FAD mice (“AD unique”), and an overlap of 4,332 proteins shared by both groups (“Overlay”, Fig. 2D). Among common genes, we identified 1757 down-regulated and 70 up-regulated proteins in 5×FAD samples compared to the WT group, with a total of 458 up-regulated and 2472 down-regulated proteins identified when including unique protein subsets (Fig. 2E-F). However, due to the limited number of up-regulated genes, no significant KEGG pathways were enriched (Fig. 2G). In contrast, down-regulated proteins in the 5×FAD group were associated with a variety of biological processes and pathways, including KEGG pathways related explicitly to neurodegenerative diseases, such as “Pathways of Neurodegeneration-Multiple Diseases” and “Alzheimer’s Disease” (Fig. 2H). We performed an analysis of enriched cell types associated with ‘AD down’ proteins using data from the single-cell RNA sequencing database (Dropviz, https://dropviz.org). Our results showed that the majority (72.27%) of downregulated proteins were predominantly expressed in excitatory neurons, while 23.13% were enriched in GABAergic interneurons. A smaller fraction (4.6%) was associated with glial cells (Fig. 2I). These results highlight significant molecular changes in the 5×FAD hippocampus, primarily driven by the downregulation of proteins associated with neurodegeneration. This highlights early proteomic changes that may contribute to the progression of AD.

### Minimal mRNA changes occur in the early-stages of Alzheimer’s disease model

**Fig. 3:**
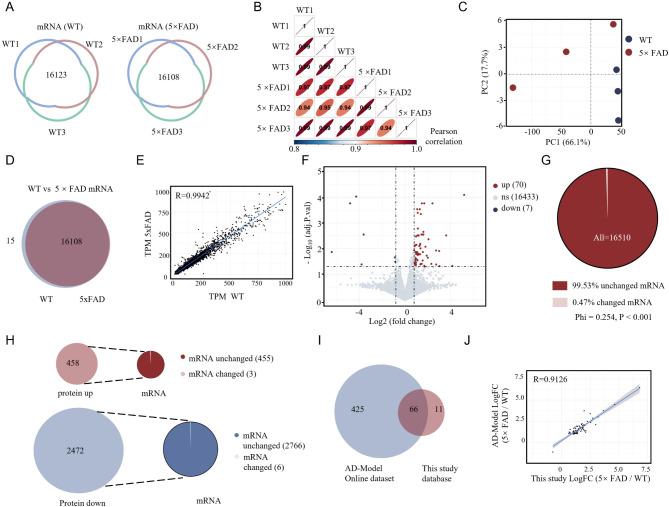
Transcriptomic profiling of hippocampal mRNA in 3-month-old WT and 5×FAD mice shows that the expression profiles mainly overlap with only minimal differences. **A** RNA-sequencing was performed on three independent samples from each group. A total of 16,123 mRNAs (raw count > 1) were detected in at least two WT samples, and 16,108 mRNAs were detected in the 5×FAD group. Non-overlapping regions indicate mRNAs detected only in isolated samples. **B** Correlation analysis demonstrated strong intra-group and inter-group consistency, visualized through an ellipse plot where Spearman’s correlation coefficients are shown in black. Flatter ellipses indicate stronger correlations. **C** Based on the PCA of the top 50% most variable mRNAs, normalized by Transcripts Per Million (TPM), there was no clear separation between the WT (blue dots) and 5×FAD (red dots) samples along PC1, suggesting overlapping transcriptomic profiles between the two groups. **D** A Venn diagram illustrating the overlap of mRNAs detected in both WT and 5×FAD mice, revealing 16,108 shared mRNAs, with 15 unique to WT. **E** Scatter plot comparing average TPM values for mRNAs between WT (x-axis) and 5×FAD (y-axis) groups, showing high overall correlation (*R* = 0.9942), indicating largely conserved expression levels. **F** Volcano plot of mRNA expression differences, indicating that 16,433 mRNAs showed no significant change. **G** Pie chart depicting the percentage of differentially expressed mRNAs versus non-significant mRNAs in 5×FAD compared to WT, from a total of 16,510 mRNAs. **H** Sector graph showing the number of differentially expressed mRNAs versus non-significant mRNAs related to upregulated and downregulated proteins. Only 3 up-regulated and 6 down-regulated proteins corresponded to differentially expressed mRNAs. **I** Venn diagram illustrating the intersection of differentially expressed mRNAs between this study and an AD-model online database, revealing 66 overlapping mRNAs. **J** Scatter plot of the 66 overlapping differentially expressed mRNAs between this study (x-axis) and the AD-model online database (y-axis), showing a high correlation in fold change (*R* = 0.9126)

To assess transcriptomic changes in the hippocampus of 3-month-old WT and 5×FAD mice, we performed mRNA sequencing with three biological replicates per group, and considered results reliable if detected in at least two replicates. Approximately 96% of the mRNAs in the RNA-seq data met this reliability criterion. A total of 16,237 reliable mRNAs were identified in the WT group and 16,108 in the 5×FAD group (Fig. 3A). Correlation analysis showed a high degree of consistency both within and between groups, as evidenced by the high Spearman’s correlation coefficients (Fig. 3B). PCA of the top 50% most variable mRNAs indicated that clustering differences between the WT and 5×FAD groups were subtle (Fig. 3C). Most mRNAs were shared between the two groups, with 16,108 overlapping transcripts, while 15 were unique to WT (Fig. 3D). A scatter plot of TPM means for mRNA expression levels showed a strong correlation (*R* = 0.9942), indicating that expression patterns were largely conserved between the two groups (Fig. 3E). Despite this overall similarity, differential expression analysis using edgeR identified a limited number of specific changes that met the criteria of an adjusted p-value of less than 0.05 and a fold change greater than 2 (Fig. 3F). A mere 0.47% of the mRNA exhibited differential expression (Fig. 3G). Specifically, three mRNA were found to be differentially expressed in association with up-regulated protein, while six mRNA were found to be differentially expressed in association with down-regulated protein (Fig. 3H). Cross-referencing these differentially expressed mRNAs with an AD model database (AD Knowledge Portal, https://adknowledgeportal.synapse.org/) revealed 66 overlapping transcripts, consistent with previously reported 5×FAD RNA-seq data and confirming the reliability of our results, as shown by a high correlation in fold change (*R* = 0.9126) (Fig. 3I-J). In conclusion, although the global mRNA expression patterns in 5×FAD mice were similar to those of WT controls, a small subset of differentially expressed transcripts may play a critical role in initiating early molecular changes during the early-stages of AD. However, this also means that the significant changes observed in the proteome of 3-month-old 5×FAD mice may not be entirely due to changes in mRNA expression. Factors not differentially expressed at the mRNA level may influence protein expression, highlighting the importance of post-transcriptional modifications as key elements in the early pathophysiological processes of AD.

### Comparative ac4C RIP sequencing profiling in hippocampal tissue from WT and 5×FAD mice

**Fig. 4:**
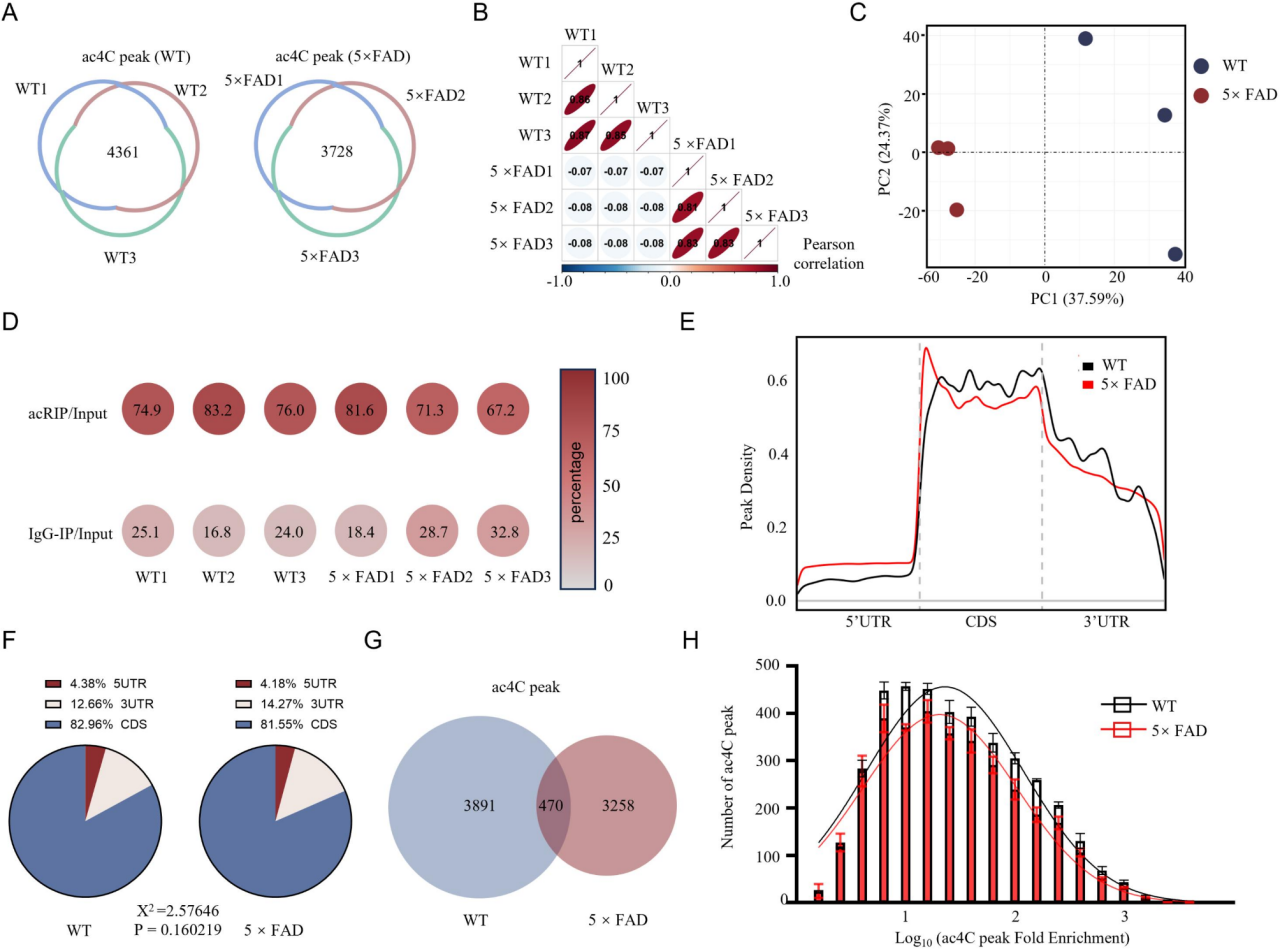
acRIP-seq analysis reveals distinct ac4C modification profiles in the hippocampus of 3-month-old WT and 5×FAD mice. **A** acRIP-seq performed on three independent samples from each group identified 4,361 ac4C peaks in WT and 3,728 peaks in 5×FAD, with non-overlapping regions representing peaks unique to specific samples. **B** Correlation analysis revealed strong within-group consistency but weaker between-group correlations, visualized with an ellipse plot where flatter ellipses corresponded to stronger correlations, as indicated by Spearman’s coefficients in black. **C** A total of 7,619 ac4C peaks were identified in both groups. PCA of the top 50% most variable peaks by MAD showed a clear separation between WT (blue dots) and 5×FAD (red dots), indicating distinct ac4C profiles. **D** The proportion of ac4C peaks detected after acRIP or IgG-IP is shown, with the color intensity representing the percentage contribution to the total number of peaks. **E-F** The distribution of ac4C peaks was analyzed, and it was found that they are present in different transcript regions (Fig. E) and genome segments, indicating that ac4C modifications are widely distributed. It is noteworthy that approximately 80% of the ac4C modifications were located in the CDS region in both the WT and 5×FAD groups (Fig. F). **G** Venn diagram highlighted 470 shared ac4C peaks, whereas 3,891 peaks were unique to WT and 3,258 were unique to 5×FAD, highlighting significant group-specific ac4C changes. **H** Histogram depicting the frequency distribution of FE in WT and 5×FAD mice, showing natural Log_10_ transformed FE (Log_10_ FE) values. The fitted curve showed a lower area under the curve for 5×FAD (Area = 707.4, SD = 0.7098) compared to WT (Area = 827.8, SD = 0.7239), with one-way ANOVA confirming a significant difference in peak counts (**P < 0.01)

To investigate differences in ac4C modification in the hippocampus of 3-month-old WT and 5×FAD mice, we performed acRIP-seq on three independent samples per group. In the WT group, 4,361 ac4C peaks were identified (fold enrichment > 1, adjusted p-value < 0.05), with peaks considered reliable if detected in at least two replicates and located on mRNAs validated by RNA-seq. In contrast, the 5×FAD group showed 3,728 ac4C peaks. The detection of peaks in individual samples demonstrated strong reproducibility across samples (Fig. 4A). The intra-group correlation analysis revealed a high degree of consistency within the WT and 5×FAD groups, with weaker correlations between the groups, indicating the presence of distinct ac4C modification patterns in each group. This was corroborated by the PCA analysis, which revealed a clear distinction between the WT and 5×FAD samples (Fig. 4B-C). The analysis demonstrated that the proportion of ac4C peaks was greater in acRIP samples than in IgG-IP controls, indicating the specific enrichment of ac4C modifications (Fig. 4D). The ac4C peaks were distributed across a wide range of transcriptomic and genomic regions, suggesting that ac4C modifications are pervasive and likely play a significant role in gene regulation (Fig. 4E-F). A substantial number of group-specific peaks were observed, with 470 ac4C peaks shared between WT and 5×FAD, while 3,891 peaks were unique to WT and 3,258 to 5×FAD (Fig. 4G). The results showed that the 5×FAD group had a lower frequency distribution of FE than WT, with significant differences in peak counts (Fig. 4H). These findings suggest that ac4C modifications may be dynamically regulated in response to the AD-like pathology of the 5×FAD model, potentially influencing the gene expression landscape in the early-stages of the disease.

### Characterization of ac4C mRNAs and comparative analysis between 5×FAD and WT mice

**Fig. 5:**
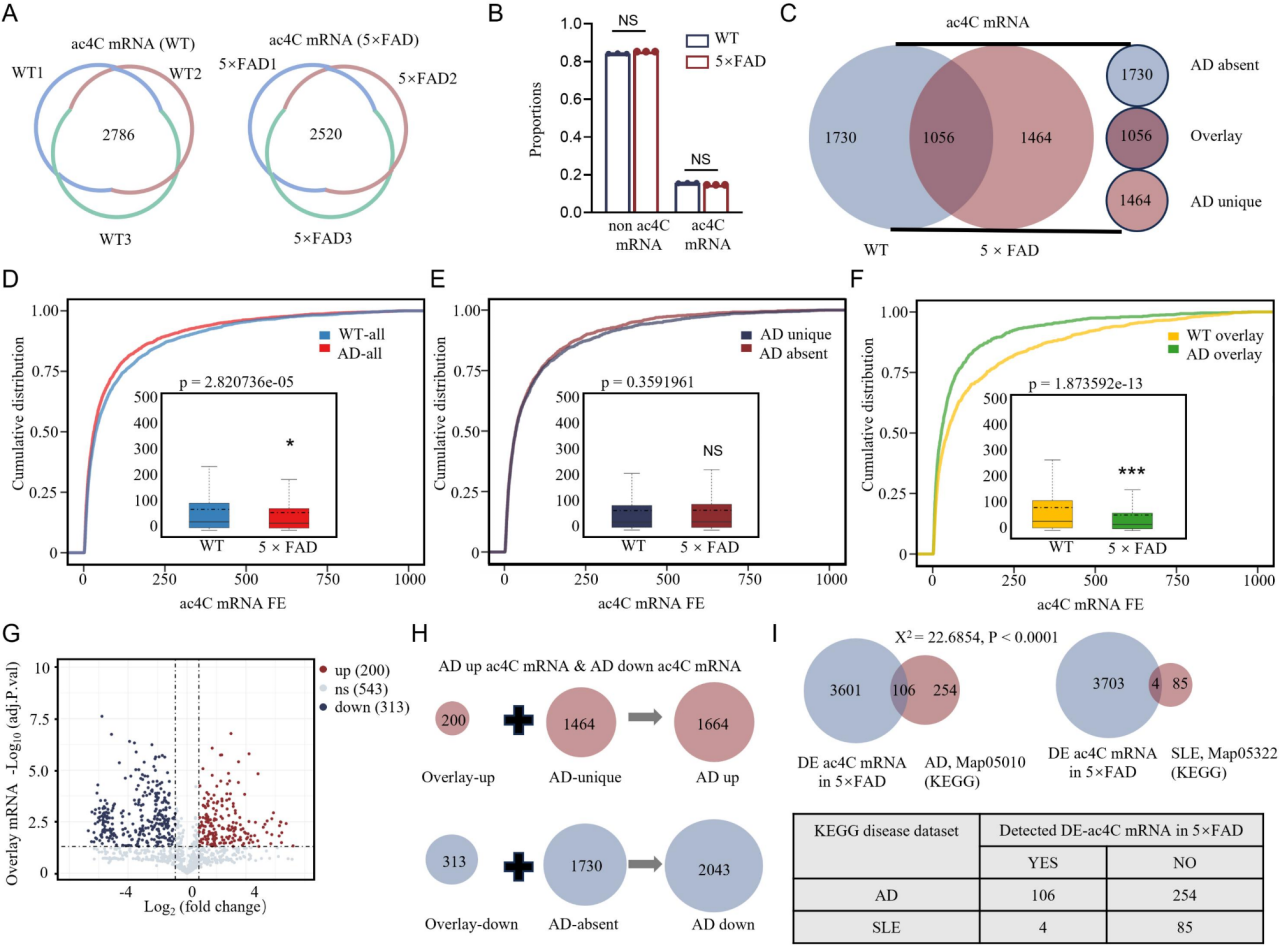
Differential analysis reveals significant changes in ac4C-modified mRNAs between WT and 5×FAD mice. **A** A total of 2,786 ac4C-modified mRNAs were identified in at least two WT samples, while 2,520 were detected in 5×FAD samples, with all transcripts confirmed by RNA-seq. **B** Bar graph comparing the ratio of ac4C-modified to non-ac4C mRNAs showed no significant difference between WT and 5×FAD mice (*P* > 0.99 for both comparisons). **C** The Venn diagram revealed 1,056 common ac4C-modified mRNAs (“Overlay”), 1,730 unique to WT (“AD absent”), and 1,464 unique to 5×FAD (“AD unique”). “AD absent” represents mRNAs found only in WT mice, “Overlay” indicates shared mRNAs, and “AD unique” refers to mRNAs present only in 5×FAD mice. **D-F** Cumulative distribution plots showed differential fold enrichment of ac4C mRNAs between groups, with quartile box plots showing medians (solid lines) and means (dashed lines). **D** Comparison of ac4C mRNA between WT and 5×FAD group (*P < 0.05). **E** No significant difference was observed between “AD absent” and “AD unique” ac4C mRNAs (*P* = 0.3581). **F** A highly significant difference was found in the “overlay” ac4C mRNAs between WT and 5×FAD (***P < 0.0001). **G** A volcano plot depicted 200 up-regulated and 313 down-regulated “overlay” ac4C mRNAs in 5×FAD compared to WT, using an adjusted p-value < 0.05 and a fold change > 2 with statistical corrections. **H** Differential analysis identified 1,664 upregulated ac4C-modified mRNAs in 5×FAD mice, including 200 “overlay” and 1,464 “AD unique” mRNAs. Additionally, 2,043 downregulated ac4C-modified mRNAs were identified, comprising 313 “overlay” and 1,730 “AD unique” mRNAs. **I** KEGG disease dataset analysis revealed that 106 differentially expressed ac4C-modified mRNAs overlapped with Alzheimer’s disease-associated genes, while 4 overlapped with systemic lupus erythematosus-associated genes. The AD gene set showed a stronger association with differentially expressed ac4C-modified mRNAs compared to the SLE gene set, confirmed by a chi-squared test with Yates’ correction (***P < 0.0001)

To elucidate the distinctions in ac4C-modified mRNAs between 5×FAD and WT mice, acRIP-sequencing was performed with rigorous criteria, including only ac4C-modified mRNAs detected in at least two out of three samples per group and validated by RNA-seq. we identified a total of 2,786 ac4C-modified mRNAs in WT samples and 2,520 in 5×FAD samples (Fig. 5A). No significant difference was observed in the proportion of ac4C-modified to non-ac4C mRNAs between the groups (Fig. 5B). The Venn analysis demonstrated that, while WT and 5×FAD shared a considerable number of ac4C modified mRNAs, there were also unique mRNAs identified in each group. The “AD absent” category was exclusive to the WT group, while the “overlay” category was common to both groups. The “AD unique” category, however, was specific to the 5×FAD group (Fig. 5C). Cumulative distribution plots revealed disparities in the fold enrichment (FE) of ac4C-modified mRNAs, particularly for the “overlay” group. In contrast, no notable discrepancy was observed for “AD absent” and “AD unique” mRNAs (Fig. 5D-F). A direct comparison of the “overlay” category revealed that 200 ac4C-modified mRNAs were upregulated and 313 were downregulated in 5×FAD relative to WT, as defined by an adjusted p-value of less than 0.05 and a fold change greater than 2 (Fig. 5G). The 5×FAD group exhibited 1,664 upregulated ac4C-modified mRNAs, including 200 common “overlay” mRNAs and 1,464 “AD unique” mRNAs specific to 5×FAD. In addition, 2,043 downregulated ac4C-modified mRNAs were identified, including 313 “overlay” mRNAs and 1,730 “AD unique” mRNAs (Fig. 5H). KEGG pathway analysis (KEGG DISEASE Database) indicated significant overlap between the differentially expressed ac4C-modified mRNAs in 5×FAD and genes linked to Alzheimer’s disease (Map05010), with 106 shared genes (Fig. 5I). These findings indicate that while the overall proportion of ac4C-modified mRNAs remains similar between WT and 5×FAD mice, specific mRNAs exhibit altered ac4C enrichment and expression in 5×FAD, particularly those associated with AD-related pathways. This observation suggests the potential involvement of targeted ac4C modifications in the early transcriptomic dysregulation of AD, and thus warrants further investigation into their functional implications and contributions to disease progression.

### Integrated analysis of differentially expressed ac4C mRNAs and proteins in 5×FAD mice identifys early Alzheimer’s disease-associated genes

**Fig. 6:**
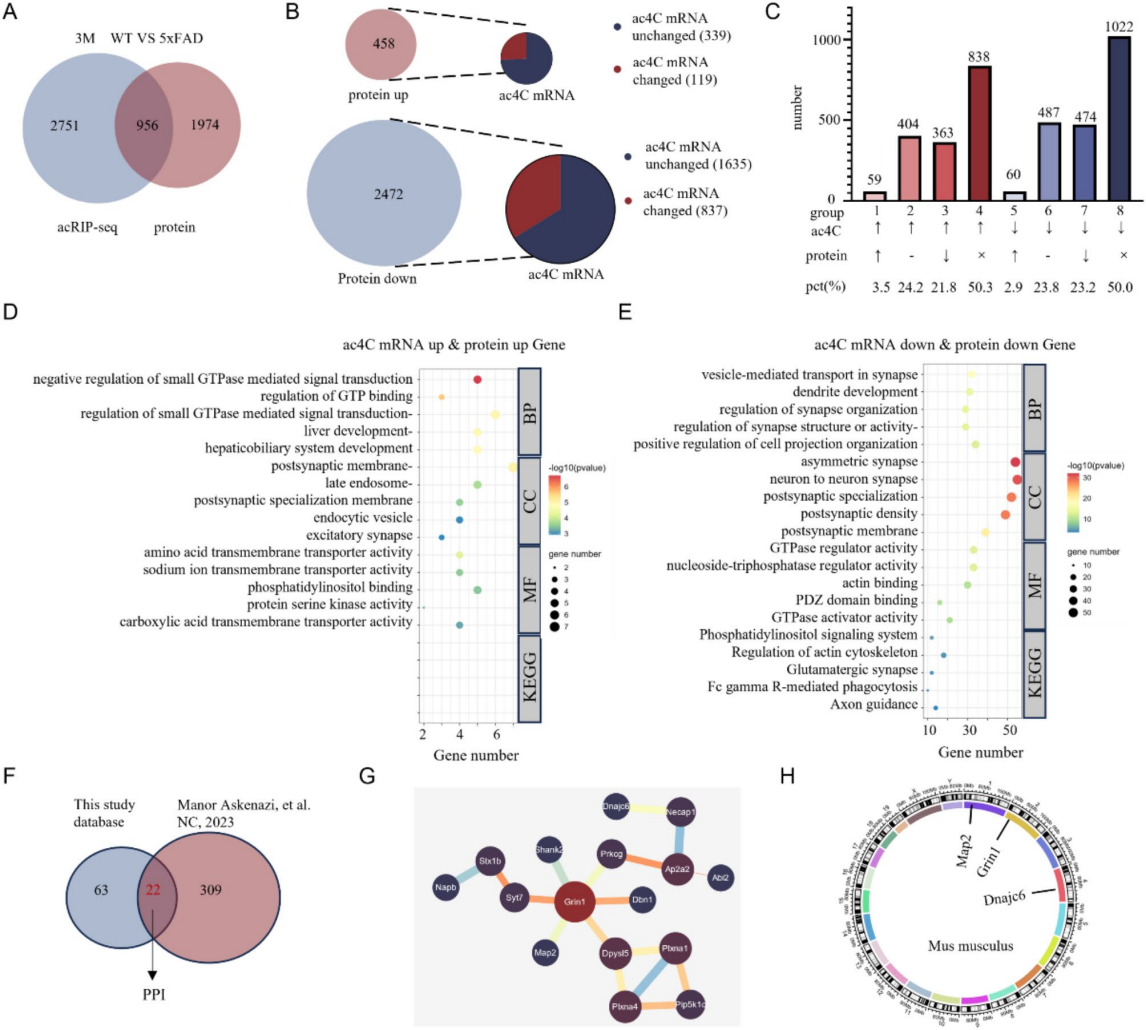
Integrated analysis highlights significant overlaps and pathway associations between proteomics and acRIP-seq data in WT and 5×FAD mice. **A** Venn diagram revealed that 956 genes exhibited differential expression at both the ac4C mRNA and protein levels. **B** Sector plot showing the number of differentially expressed versus non-significantly expressed ac4C-modified mRNAs associated with upregulated and downregulated proteins. A total of 119 upregulated and 837 downregulated proteins corresponded to differentially expressed ac4C-modified mRNAs. **C** Histogram showing the regulation of proteins corresponding to up- and downregulated ac4C-modified mRNAs. **D-E** GO and KEGG pathway enrichment analysis for group 1 (upregulated ac4C-modified mRNAs and proteins) and group 7 (downregulated ac4C-modified mRNAs and proteins). The top 5 BP, MF, CC, and KEGG pathways were summarized for group 1 (D). Similar analyses for group 7 are shown in (E), with an adjusted p-value threshold of < 0.05. **F** Venn diagram showing the intersection of downregulated genes between the top 5 BPs from (E) and data from a human hippocampal proteomic study, identifying 22 overlapping genes. **G** Interaction network of the 22 overlapping genes identified in (F), constructed using STRING analysis. Nodes in the network are color-coded from red to blue, indicating genes with low to high levels of connectivity, ***P < 0.0001. **H** Circos plot showing the chromosomal localization of hub genes selected based on their connectivity degrees and biological functions. Glutamate ionotropic receptor NMDA type subunit 1, Grin1; Microtubule associated protein 2, Map2; DnaJ heat shock protein family (Hsp40) member C6, Dnajc6

To assess early molecular differences in 3-month-old WT and 5×FAD mice, we performed a comprehensive analysis integrating proteomic and acRIP-seq data. A Venn diagram revealed 956 genes that were differentially expressed at both the ac4C mRNA and protein levels, suggesting a coordinated transcriptomic and proteomic dysregulation in the early-stages of AD pathology in 5×FAD mice (Fig. 6A). A sector plot revealed that 119 up-regulated and 837 down-regulated proteins corresponded to differentially expressed ac4C-modified mRNAs (Fig. 6B). The histogram showed the protein expression of the differentially expressed ac4C-modified mRNAs (Fig. 6C). Those suggested the potential influence of ac4C modifications in post-transcriptional regulatory mechanisms affecting protein expression. Our combined analysis of multi-omics data showed compared with mRNA expression differential, mRNA ac4C-modified expressed differential was more likely to participate in sharp protein differential expression of 3-month-old 5×FAD mice. Arango’s research demonstrated that the ac4C modification in the CDS region of mRNA can enhance protein translation [32]. In this study, approximately 80% of the acetylation modifications were observed to occur within the CDS region of mRNA. Accordingly, we selected groups in which alterations in protein expression and ac4C modifications were uniform for subsequent enrichment analysis. Group 1 (protein upregulated and ac4C mRNA upregulated) and Group 7 (protein downregulated and ac4C mRNA downregulated). GO and KEGG pathway enrichment analyses of upregulated (group 1) and downregulated (group 7) ac4C-modified mRNAs and proteins revealed distinct biological processes and pathways. Group 1 was enriched for pathways related to cellular and molecular functions (Fig. 6D), whereas group 7 showed significant associations with neurodegenerative processes, including proteins essential for synaptic function (Fig. 6E). Cross-referencing with human hippocampal proteomic data [33] identified 22 downregulated genes common to group 7 and human studies, indicating the conservation of dysregulated pathways in early AD (Fig. 6F). STRING analysis of these 22 genes revealed an interaction network with varying degrees of connectivity, suggesting potential hub genes that may act as key regulators in early AD (Fig. 6G). Chromosomal mapping using a circos plot illustrated the distribution of these hub genes, highlighting their potential role in early AD mechanisms (Fig. 6H). These findings suggest the contribution of differential ac4C modifications to early transcriptomic and proteomic changes associated with AD and support their relevance as targets for future research on AD pathogenesis.

### Reduced ac4C epigenetic marks and protein levels of key neuronal proteins in the 5×FAD Alzheimer’s model

**Fig. 7:**
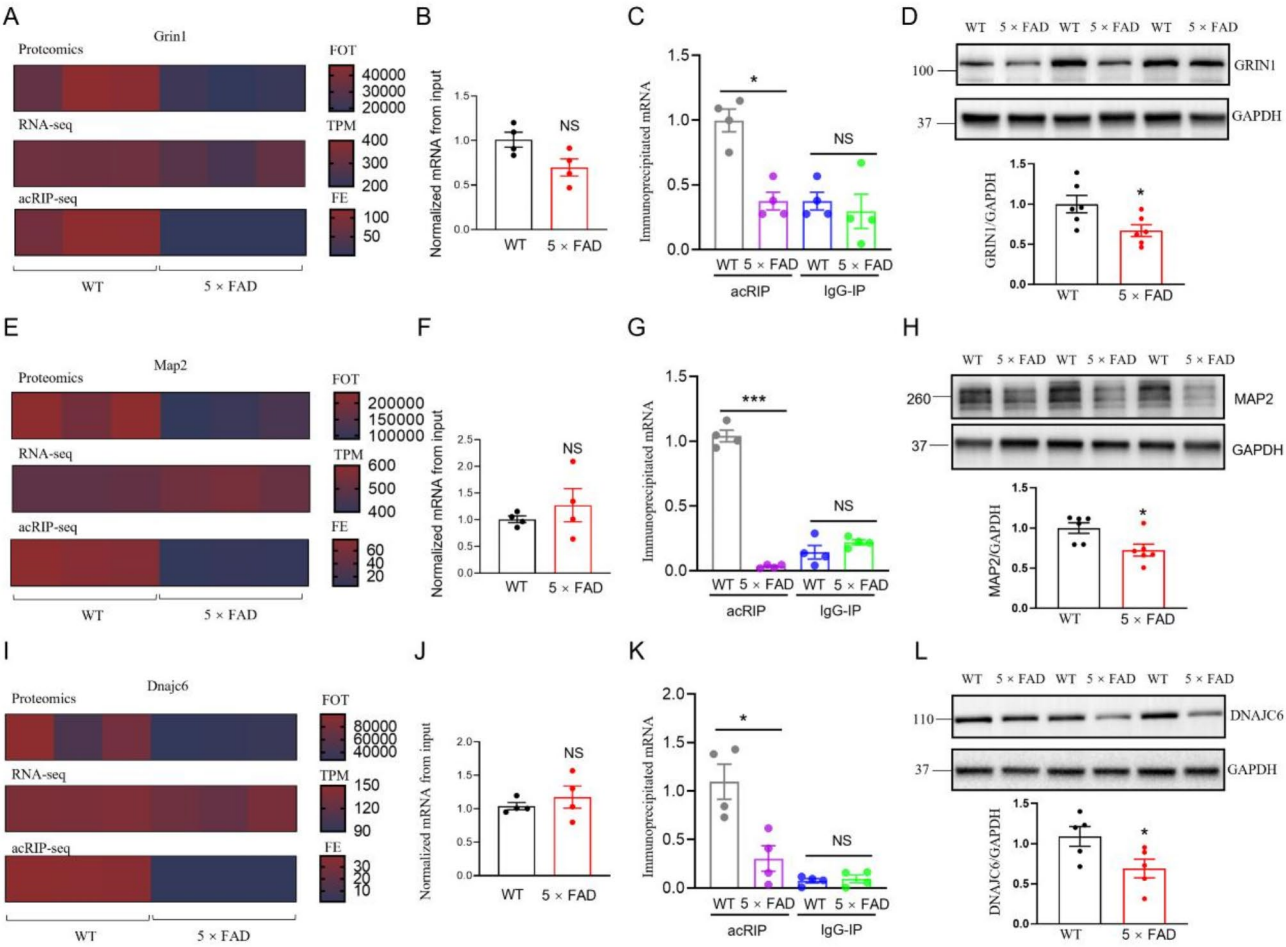
Significant reduction in ac4C modification and protein levels of GRIN1, MAP2, and DNAJC6 was evident in the hippocampus of 5×FAD mice. **A** Heatmap of gene expression across Proteomics, RNA-seq, and acRIP-seq for Grin1 between 5×FAD mice and matched WT control samples, where red indicates higher expression and blue indicates lower expression. **B** The expression of Grin1 in the input samples shows no significant differences between WT and 5×FAD mice after normalization (NS, not significant, two-tailed t-test, *n* = 4 biological replicates per condition). **C** RT-qPCR analysis of acRIP and IgG-IP samples from WT and 5×FAD mice reveal a significant reduction in the acetylation modification of Grin1 in 5×FAD mice (*P = 0.0282, NS for no significant difference, two-tailed t-test, *n* = 4 biological replicates per condition). **D** Western blot analysis shows lower protein expression levels of GRIN1 in the hippocampus of 3-month-old 5×FAD mice compared to WT controls. Top: Representative western blot images; bottom: Quantification of protein levels. *P = 0.03, two-tailed t-test, *n* = 6 biological replicates per condition. Data were normalized to WT. **E** Heatmap of gene expression across Proteomics, RNA-seq, and acRIP-seq for Map2 between 5×FAD mice and matched WT control samples, where red indicates higher expression and blue indicates lower expression. **F** The expression of Map2 in the input samples shows no significant differences between WT and 5×FAD mice after normalization (NS, not significant, two-tailed t-test, *n* = 4 biological replicates per condition). **G** RT-qPCR analysis of acRIP and IgG-IP samples from WT and 5×FAD mice reveal a significant reduction in the acetylation modification of Grin1 in 5×FAD mice (***P = 0.0002, NS for no significant difference, two-tailed t-test, *n* = 4 biological replicates per condition). **H** Western blot analysis shows lower protein expression levels of MAP2 in the hippocampus of 3-month-old 5×FAD mice compared to WT controls. Top: Representative western blot images; bottom: Quantification of protein levels. *P = 0.0201, two-tailed t-test, *n* = 6 biological replicates per condition. Data were normalized to WT. **I** Heatmap of gene expression across Proteomics, RNA-seq, and acRIP-seq for Dnajc6 between 5×FAD mice and matched WT control samples, where red indicates higher expression and blue indicates lower expression. **J** The expression of Dnajc6 in the input samples shows no significant differences between WT and 5×FAD mice after normalization (NS, not significant, two-tailed t-test, *n* = 4 biological replicates per condition). **K** RT-qPCR analysis of acRIP and IgG-IP samples from WT and 5×FAD mice reveal a significant reduction in the acetylation modification of Dnajc6 in 5×FAD mice (*P = 0.0379, NS for no significant difference, two-tailed t-test, *n* = 4 biological replicates per condition). **L** Western blot analysis shows lower protein expression levels of DNAJC6 in the hippocampus of 3-month-old 5×FAD mice compared to WT controls. Top: Representative western blot images; bottom: Quantification of protein levels. *P = 0.0455, two-tailed t-test, *n* = 6 biological replicates per condition. Data were normalized to WT

To further explore the role of ac4C modification in GRIN1, MAP2, and DNAJC6 in 5×FAD mice, we compared gene and protein profiles between 5×FAD and WT control mice. First, heat map analysis revealed differences in gene expression for Grin1, Map2, and Dnajc6 across proteomics, RNA-seq, and acRIP-seq data (Fig. 7A, E, I). The expression of these genes was lower in the 5×FAD group than in the WT group as shown in the heatmaps, where red indicates higher expression and blue indicates lower expression. However, there were no significant differences in the input samples between WT and 5×FAD mice for these genes (Fig. 7B, F, J). Next, RT-qPCR analysis of acRIP and IgG-IP samples revealed a significant reduction in acetylation modification of Grin1, Map2, and Dnajc6 in the 5×FAD mice (Fig. 7C, G, K). Western blot analysis also revealed lower GRIN1, MAP2, and DNAJC6 protein levels in the hippocampus of 3-month-old 5×FAD mice compared to WT controls (Fig. 7D, H, L). These findings suggest that reduced ac4C modification and protein expression of these key proteins in the hippocampus may contribute to the early pathophysiology of AD.

### Cellular localization of key neuronal proteins in the 5×FAD Alzheimer’s model

**Fig. 8:**
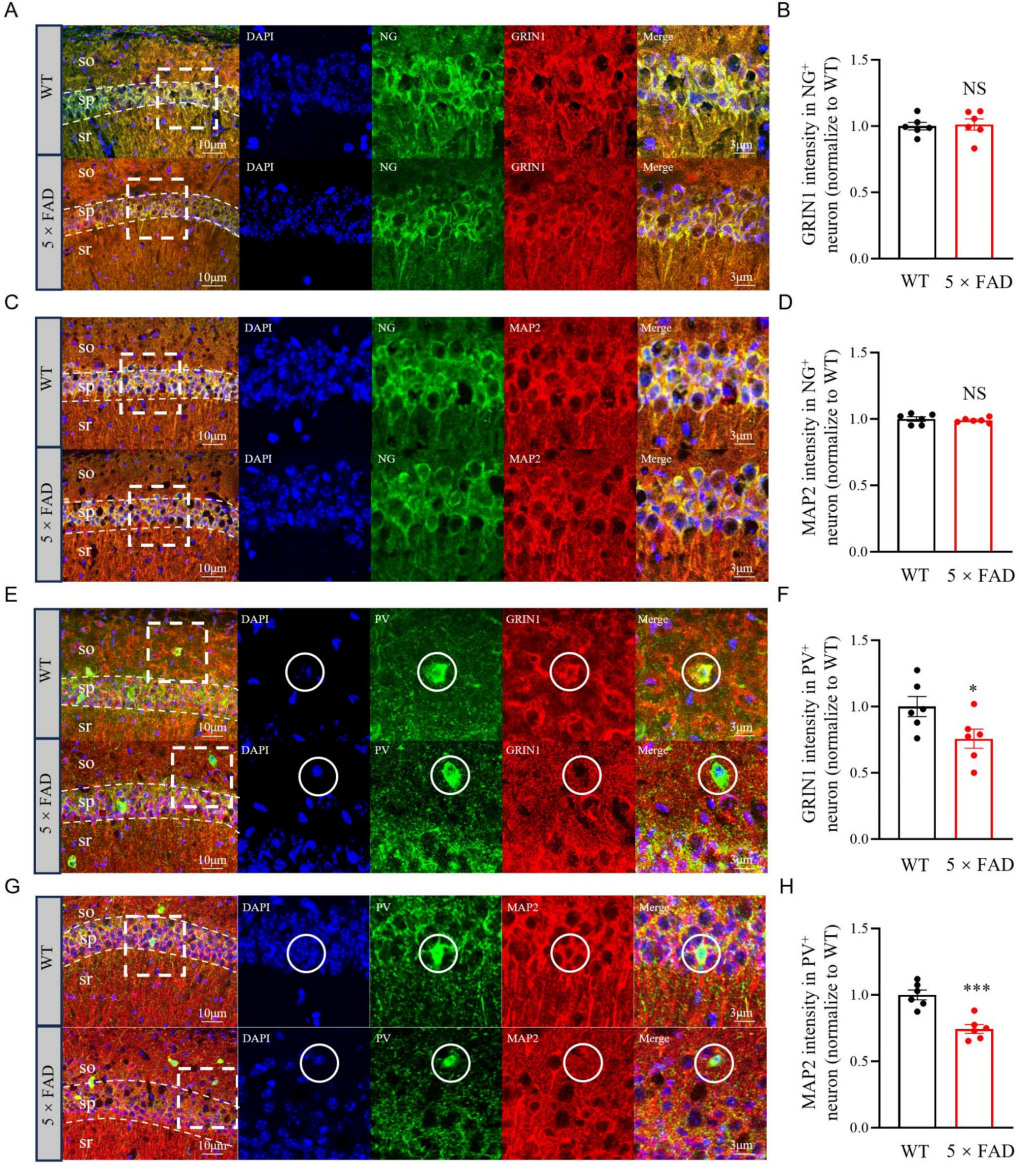
Reduction in protein levels of GRIN1 and MAP2 in GABAergic interneurons of 3-month-old 5×FAD mice. **A-B** Immunofluorescence for neurogranin (NG) and GRIN1 in the CA1 area of the hippocampus. Fluorescence intensity normalized to WT group of GRIN1 in NG-positive pyramidal neurons shows no significant difference between WT and 5×FAD mice. NS for no significant difference. **C-D**. Immunofluorescence for NG and MAP2 in the CA1 area of the hippocampus. Fluorescence intensity normalized to WT group of MAP2 in NG-positive pyramidal neurons shows no significant difference between WT and 5×FAD mice. **E-F**. Immunofluorescence for parvalbumin (PV) and GRIN1 in the CA1 area of the hippocampus, with quantification of fluorescence intensity normalized to WT group of GRIN1 in PV-positive GABAergic interneurons. A significant reduction in GRIN1 expression in PV-positive neurons is observed in the 5×FAD group compared to the WT group. *P = 0.0433. **G-H**. Immunofluorescence for PV and MAP2 in the CA1 area of the hippocampus, with quantification of fluorescence intensity normalized to WT group of MAP2 in PV-positive GABAergic interneurons. A significant reduction in MAP2 expression in PV-positive neurons is observed in the 5×FAD group compared to the WT group. ***P = 0.0004. All statistical analyses were performed using two-tailed t-test. Scale bars: left, 10 μm; right, 3 μm. Top panel: WT mouse; bottom panel: 5×FAD mouse. Abbreviations: so, stratum oriens; sp, stratum pyramidale; sr, stratum radiatum

In our investigation of key neuronal protein localization, we co-labeled Grin1 and MAP2 with specific cellular markers. However, the IF staining of DNAJC6 encountered suboptimal results due to significant background interference. We utilized the excitatory neuron marker neurogranin (NG) for simultaneous fluorescence labeling with Grin1 and MAP2. This approach revealed that there was no significant difference in the expression of Grin1 in NG-positive pyramidal neurons between WT and 5×FAD groups (Fig. 8A-B). Comparable outcomes were noted when NG was co-labeled with MAP2 (Fig. 8C-D). Considering the critical role of excitatory/inhibitory (E/I) imbalance, driven by impaired interneuron function, in the early stages of AD, we pursued deeper analysis. CIBERSORT analysis showed that 23.13% of the down-regulated proteins were predominantly found in GABAergic interneurons. Despite hippocampal interneurons constituting a minor portion of the total neuronal population, we conducted additional fluorescence co-labeling experiments using the interneuron marker parvalbumin (PV) together with Grin1 and MAP2. It was noteworthy that the expression of Grin1 on PV-positive GABAergic interneurons was significantly decreased in the 5×FAD group (Fig. 8E-F), a trend that was also evident when MAP2/PV co-labeling (Fig. 8G-H). These results indicate that MAP2 and GRIN1 are primarily expressed in excitatory neurons, but their reduction in the early stages of AD predominantly occurs in interneurons, potentially impairing interneuron function and leading to excessive neuronal hyperactivity, which contributes to AD progression.

### Distinct patterns of ac4C mRNA modifications in 3 and 6-month-old 5×FAD mice

**Fig. 9:**
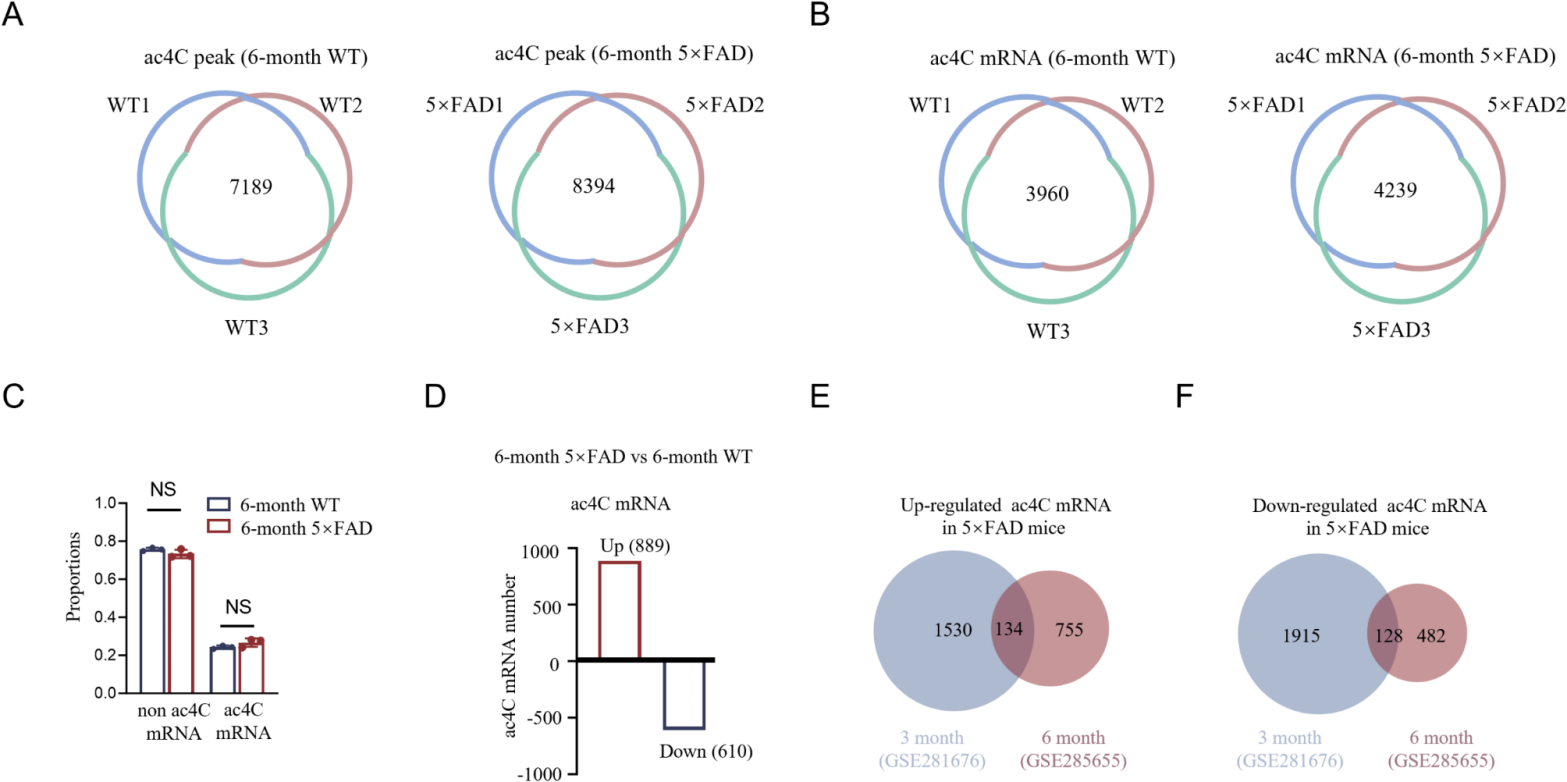
Dysregulated ac4C mRNAs identified in 3-month-old 5×FAD mice are specific to early stage of AD. **A** acRIP-seq performed on three independent samples from each group identified 7,189 ac4C peaks in 6-month-old WT and 8,394 peaks in 6-month-old 5×FAD, with non-overlapping regions representing peaks unique to specific samples. **B** A total of 3,960 ac4C-modified mRNAs were identified in at least two 6-month-old WT samples, while 4,239 were detected in 6-month-old 5×FAD samples, with all transcripts confirmed by RNA-seq. **C** Bar graph comparing the ratio of ac4C to non-ac4C mRNAs showed no significant difference between 6-month-old WT and 6-month-old 5×FAD mice (*P* > 0.99 for both comparisons). **D** A total of 889 ac4C mRNAs were upregulated, while 610 ac4C mRNAs were downregulated in 6-month-old 5×FAD mice. **E** The Venn diagram shows the overlap and distribution of ac4C mRNAs upregulated in 3 and 6-month-old 5×FAD mice compared to age-matched WT controls. A total of 134 ac4C mRNAs were upregulated in both 3-month-old and 6-month-old 5×FAD mice. In contrast, 1530 ac4C mRNAs were uniquely upregulated in 3-month-old 5×FAD mice and 755 ac4C mRNAs were uniquely upregulated in 6-month-old 5×FAD mice. **F** The Venn diagram shows the overlap and distribution of ac4C mRNAs downregulated in 3 and 6-month-old 5×FAD mice compared to age-matched WT controls. A total of 128 ac4C mRNAs were downregulated in both 3 and 6-month-old 5×FAD mice. In contrast, 1915 ac4C mRNA were uniquely downregulated in 3-month-old 5×FAD mice and 482 ac4C mRNA were uniquely downregulated in 6-month-old 5×FAD mice. On the basis of adjusted p value < 0.05,|Log_2_(FC)|> 1

In order to further explore the role of ac4C modifications on mRNA at different stages of AD, we performed on ac4C modifications in the hippocampus of 6-month-old 5×FAD and WT mice. The analysis yielded a total of 7,189 ac4C peaks in the hippocampus of 6-month-old WT mice, compared to 8,394 peaks in 6-month-old 5×FAD mice (Fig. 9A). Furthermore, 3,960 ac4C-modified mRNAs were detected in WT mice, while 4,239 were observed in 5×FAD mice (Fig. 9B). However, the proportion of ac4C-modified mRNAs relative to non-ac4C-modified mRNAs showed no significant difference between the two groups (Fig. 9C). Variance analysis showed that a total of 889 ac4C mRNAs were found to be upregulated, while 610 ac4C mRNAs were downregulated in 6-month-old 5×FAD mice (Fig. 9D). Venn diagram analysis further demonstrated distinct age-related dynamics in ac4C-modified mRNAs. In 3-month-old 5×FAD mice, 1,530 mRNAs were uniquely upregulated and 1,915 were uniquely downregulated, while only 134 upregulated and 128 downregulated mRNAs overlapped between 3 and 6-month-old 5×FAD mice (Fig. 9E-F). This finding indicates that approximately 90% of dysregulated ac4C-modified mRNAs in 3-month-old 5×FAD mice were not observed at 6 months. The results of this study suggest that the dysregulated ac4C modifications identified in 3-month-old 5×FAD mice are highly specific to the early-stages of AD, highlighting a distinct molecular signature at this critical point in disease progression.

## Discussion

It is of paramount importance to gain insight into the early molecular and epigenetic alterations associated with AD in order to elucidate the underlying mechanisms that precede the onset of clinical symptoms. The results of this study indicate that the observed alterations in protein expression in 3-month-old 5×FAD mice are unlikely to be the result of changes in mRNA abundance. It is therefore plausible that these alterations are linked to ac4C modifications, which represent a form of post-transcriptional regulation of mRNA. This emphasizes the functional importance of ac4C mRNA modifications and indicates their potential role in the initial stages of AD pathophysiology, as demonstrated by the 5×FAD mouse model. We focused on ac4C modifications because of their recognized function in stabilizing mRNA and enhancing translation, processes that are critical for neuronal health and synaptic function [6, 12, 17, 34]. Widespread perturbations in RNA homeostasis play a critical role in neuronal dysfunction [35]. The targeting and reversal of aberrant RNA modifications, including those involving ac4C, may represent a promising strategy for modulating the progressive decline observed in neurodegenerative diseases. Correcting these RNA modifications may enable the modulation of disease progression, thereby offering meaningful therapeutic benefits to individuals affected by such conditions.

The results presented in this study provide valuable insights into the role of mRNA acetylation modifications in the 5×FAD model at three months of age, supported by rigorous within-group reproducibility and stringent quality control standards. Although no significant cognitive deficits were observed in 5×FAD mice at this stage, we detecting significant changes in mRNA acetylation. To ensure the reliability of these findings, extensive quality control measures were implemented to maintain the integrity and accuracy of the data analysis. Measures included minimizing rRNA interference and validating RNA integrity and library quality, contributing to highly consistent sequencing results across biological replicates. Our quality control strategy resulted in robust data, allowing confident identification of 4,537 out of 5,373 ac4C peaks in the WT group and 3,904 out of 4,810 peaks in the 5×FAD group, with a high degree of overlap between replicates. This consistency underlines the credibility of our results and highlights the need for stringent quality standards when investigating post-transcriptional modifications such as ac4C. This foundation supports the exploration of the broader implications of ac4C modifications on mRNA stability and translation, contributing to our understanding of their potential involvement in the early pathophysiology of AD.

To validate the identification of ac4C peaks, we ensured corroboration with RNA-seq data, confirming the presence of the associated mRNA and reinforcing our findings’ credibility. By employing this rigorous approach, 4,361 credible peaks were confirmed in the WT group and 3,728 in the 5×FAD group. Notably, the 5×FAD group exhibited 1,464 newly acetylated mRNAs and a loss of acetylation in 1,730 mRNAs, compared to 2,789 acetylated mRNAs in age-matched WT mice. Given the well-established influence of ac4C modifications on mRNA translation and stability, it is plausible that these pronounced changes contribute to the pathophysiological processes observed in 3-month-old 5×FAD mice. Notably, that the RNA-seq data revealed no correlation between the pronounced alterations in mRNA acetylation in the hippocampus and significant changes in overall mRNA expression levels. This indicates that ac4C modifications may modulate translation efficiency or influence the spatial conformation of mRNA molecules. Previous studies have demonstrated that ac4C can impact RNA binding properties, which supports this proposition [11]. This evidence supports to the hypothesis that mRNA acetylation may affect in translational regulation.

Here, the proteomic and transcriptomic data demonstrate that, while overall mRNA expression alterations were minimal in early 5×FAD mice, specific ac4C modifications exerted a pronounced influence on the proteomic landscape. The observed differential ac4C enrichment indicates that even subtle RNA modifications can disrupt protein synthesis pathways, potentially driving downstream effects that contribute to early AD pathogenesis. These findings emphasize the necessity of investigating RNA modifications as potential early modulators of AD, thereby elucidating their role in initiating the molecular changes that precede broader neurodegenerative processes. Our results indicate a reduction in protein in 5×FAD mice, which may be associated with the downregulation of the corresponding ac4C-modified mRNAs. GO analysis revealed that these genes are significantly enriched in BP related to synaptic function, including vesicle-mediated transport in synapses, dendrite development, and regulation of synapse organization, among others. It is noteworthy that synaptic dysfunction and an imbalance in excitatory and inhibitory neurotransmission are believed to emerge decades before the onset of cognitive decline. This is accompanied by the loss of presynaptic terminals, which precedes the formation of amyloid beta plaques [36, 37]. This underscores the potential for identifying the pathological mechanisms underlying synaptic dysfunction, thereby enabling early diagnosis and intervention for individuals at high risk of AD, even prior to cognitive decline or Aβ plaque formation. The present study demonstrates that in the early-stages of AD, the expression of proteins associated with synaptic function is diminished. It is noteworthy that while the levels of corresponding mRNAs show no significant differences, the ac4C modification of these mRNAs is markedly downregulated. This suggests that ac4C modification may contribute to the pathological processes leading to altered protein expression associated with synaptic dysfunction in early-stage AD. These findings may help explain the lack of significant changes in mRNA expression observed in previous studies, underscoring the importance of investigating post-transcriptional modifications in AD pathology.

However, the overlap between the genes identified in our study and those from human sequencing data is limited, which may be explained by several factors. (1) The human sequencing data were obtained from postmortem brain tissues of middle- or late-stage AD patients, whereas the 3-month-old 5×FAD mouse model represents the early stages of the disease. (2) Mice and humans differ in gene expression patterns and disease mechanisms, which can result in discrepancies when comparing data from animal models to human studies [38, 39]. (3) Gene expression changes in AD have been shown to be highly region-specific and cell-type-specific [40]. This study focused on the hippocampus, while human postmortem studies examined a broader range of brain regions, including the entorhinal cortex, hippocampus, parahippocampal gyrus, temporal cortex, frontal cortex, parietal cortex, precuneus, cingulate, and occipital cortex.

Furthermore, an exhaustive examination was undertaken of the genes associated with these pathways, resulting in the identification of representative genes that are intimately connected with synaptic function. It is noteworthy that GRIN1, MAP2, and DNAJC6 play a critical role in maintaining synaptic structure and function. GRIN1, which encodes a subunit of the NMDA receptor, is essential for synaptic plasticity and cognitive function [41]. Memantine, an NMDA receptor antagonist, has been shown to alleviate chronic neurotoxicity and improve learning and memory by inhibiting overactivation mediated by the NR1 subunit [42]. In this regard, the downregulation of NR1 subunit of NMDA receptor (Grin1) may offer neuroprotective benefits for AD. The dysfunction of MAP2 has been demonstrated to be closely associated with the pathology of AD, including the formation of neurofibrillary tangles, synaptic dysfunction, and microtubule disassembly [43]. MAP2 is critical in stabilizing the neuronal microtubule network, which is necessary to maintain synaptic connectivity. It is noteworthy that decreased MAP2 expression at synapses precedes the appearance of AD plaques, suggesting that MAP2 alterations may serve as early markers of synaptic pathology and cognitive impairment [44]. DNAJC6 is involved in synaptic vesicle recycling and efficient neurotransmission, with mutations associated with neurodevelopmental and synaptic defects and early-onset parkinsonism [45]. Clinical studies have linked the HSP family proteins, including Dnajc6, to genetic risks of AD. Members of this family have been shown to regulate Aβ protein aggregation, thereby providing a protective effect for AD [46, 47]. The observed downregulation of DNAJC6 in 5×FAD mice suggest impaired vesicle cycling contributes to early synaptic dysfunction.

The imbalance between excitation and inhibition (E/I) is a key pathological hypothesis of AD [48]. Notably, individuals with abnormal neuronal activity, such as epilepsy patients, exhibit a significantly higher incidence of AD in old age. In 5×FAD mice with epileptic seizures, learning and memory deficits are exacerbated [49]. In our study, we observed that GRIN1 and MAP2 were significantly downregulated in PV-positive interneurons, rather than in excitatory neurons, despite interneurons constituting a small proportion of hippocampal neurons. This downregulation of key neuronal proteins likely results in impaired interneuron function, which in turn may lead to abnormal hyperactivity of excitatory hippocampal neurons. These findings point to an early-stage alteration in AD pathology. Previous studies have suggested that MAP2 expression remains unchanged in hippocampal neurons of early-stage AD model mice [50]. Our results are consistent with previous studies [51, 52], indicating that deficits in PV-positive GABAergic interneurons mediate early dysfunction of key proteins in AD. This highlights the critical value of integrative multi-omics approaches, including epigenomic and transcriptomic analyses, in uncovering early-stage pathological changes that might have been previously neglected. By focusing on these lesser-explored cellular populations, we can gain new insights into AD progression and identify potential therapeutic targets that may otherwise have been missed. This collective evidence underscores the importance of ac4C as a regulatory element in protein synthesis and RNA stability, it suggests that reductions in ac4C modifications may trigger early synaptic dysfunction that sets the stage for broader neurodegenerative processes. Moreover, Investigating the role of ac4C modifications in the hippocampus could be particularly valuable in understanding their impact on the regulation of cognitive function in AD. Disruption of these modifications may contribute to AD pathology, highlighting the potential importance of ac4C in the progression of the disease. Understanding these early transcriptomic and proteomic perturbations provides insights into potential molecular targets for therapeutic intervention in AD.

In conclusion, both proteomic and transcriptomic analyses reveal significant dysregulation of ac4C modifications and protein expression, strongly supporting an early neurodegenerative phenotype in the 5×FAD mouse model of AD. Functional enrichment analyses linked downregulated ac4C-modified mRNAs and proteins to pathways associated with neurodegeneration and AD, findings supported by overlap with human disease studies. This alignment highlights the potential contribution of ac4C modifications to early cellular dysfunctions consistent with human AD pathology. Given the current limitations of effective treatment options for AD, understanding these early pathophysiological mechanisms is essential. The focus of this study on ac4C modifications provides new insights into the early molecular changes associated with AD and suggests that these modifications may serve as critical regulators of RNA stability, protein interactions, and neuronal health. Studying these early changes may pave the way for developing novel biomarkers and targeted therapies that could help detect and intervene in the early-stages of the disease, potentially slowing or preventing its progression. Future research should extend these findings by examining the impact of altered ac4C modifications in different brain regions and in human studies. Addressing these early transcriptomic and proteomic changes may provide new opportunities for interventions that prevent or slow significant neurodegenerative damage and ultimately alter the course of AD.

### Method

#### Animal

All experimental procedures were approved by the Institutional Animal Care and Use Committee of East China Normal University (ethics approval numbers: m20220604 and m20241104). In this study, we used three-month-old female 5×FAD mice (B6.Cg-Tg [APPSwFlLon, PSEN1M146LL286V] 6799Vas/Mmjax) together with three-month-old female wild-type (WT) mice, both obtained from The Jackson Laboratory (MMRRC). All mice were housed under the same conditions: 23 °C temperature, 50% humidity, and a 12-hour light/dark cycle with ad libitum access to food and water. Three-month-old male and female littermates were used in the experiments. Animals were anesthetized with isoflurane and intracardiac perfusion with phosphate-buffered saline (PBS).

#### Morris water maze (MWM)

The Morris Water Maze (MWM) test was used to assess spatial learning and memory. The MWM apparatus consisted of a cylindrical water tank (diameter: 1.5 m, height: 50 cm) filled with water at 23 °C to a depth of 30 cm. A white plastic platform (diameter: 10 cm) was submerged 6–8 mm below the water surface. The tank was surrounded by a black opaque screen with simple graphic markers placed in each cardinal direction to aid spatial orientation. A camera mounted above the tank transmitted real-time images to a computer for recording and analysis. White, opaque, non-toxic paint was added to the water to obscure visibility. The mice were trained for five consecutive days and tested on the sixth day. On the first day, a visible marker was placed on the platform to help the mice identify its location. The tank was divided into four quadrants, with the platform in the first quadrant. Each mouse was trained four times per day, with each trial starting in a different quadrant. On days 2–5, the marker was removed from the platform. Mice were released from a new quadrant each day and trained in four daily trials, each beginning in a different quadrant. The timer for each trial started when the mouse was released. If the mouse found the platform within 60 s and remained on the platform for at least 3 s, the timer stopped and the event was recorded. If the mouse did not locate the platform within 60 s, it was gently guided to the platform and allowed to remain there for 15 s. Training sessions were conducted at the same time each day, and mice were gently dried after each trial. The platform was removed on the sixth day, and the mice were released from the third quadrant. Each mouse was allowed to swim for 60 s, after which it was retrieved. During the training days (days 1–5), the daily average latency for each mouse to reach the platform was recorded. On the sixth day (probe trial), the average swimming speed, total swimming distance, and number of times each mouse crossed the former platform area were recorded.

#### Chemical and reagents

RNA was isolated using an Automatic nucleic acids instrument (Vazyme, VNP-32P) for RT-qPCR. Methods for extraction and purification of poly(A) RNA for RNA-seq or acRIP-seq are described in the following sections. Chloroform and other common laboratory chemicals were purchased from Merck (109634, Darmstadt, Germany). Hydroxylamine was purchased from Aladdin Bio-Chem Technology Co., Ltd. (H164487). Methylene blue used for nucleic acid visualization was purchased from Abcam (M9140). Nylon membranes were purchased from Beyotime (FFN10). The RNase inhibitor was purchased from New England Biolabs (M0314S, NEB, CA, USA).

#### Western blotting (WB)

After euthanasia, hippocampal tissue from WT and 5×FAD mice were isolated and homogenized in RIPA buffer containing 50 mM Tris-HCl (pH 7.4), 150 mM NaCl, 2 mM EDTA, 1% sodium deoxycholate, 1% SDS, 1 mM PMSF, 50 mM sodium fluoride, 1 mM sodium vanadate, 1 mM DTT, and a protease inhibitor cocktail. SDS-PAGE resolved protein samples (20 µg)and then transferred to PVDF membranes (0.45 μm, PVH000210, Millipore). The membranes were blocked in TBS buffer (0.1% Tween-20 and 5% milk) for 1 h at room temperature and then incubated with primary antibodies overnight at 4 °C. After washing, the membranes were incubated with HRP-conjugated secondary antibody (goat anti-rabbit, G-21234, 1:2000, Thermo Fisher) in the same TBS buffer for 2 h at room temperature. Immunoreactive bands were visualized using the ChemiDoc™ XRS + Imaging System (Bio-Rad) with enhanced chemiluminescence (Pierce) and analyzed using ImageJ software (NIH). Primary antibodies used included anti-APP (1:2000, Merck Millipore, A8717), anti-GRIN1 (1:1000, HUABIO, ET1703-75), anti-MAP2 (1:1000, Merck Millipore, AB5622), anti-DNAJC6 (1:1000, HUABIO, HA722929), and anti-GAPDH (1:4000, HUABIO, ET1601-4).

#### Immunofluorescence (IF)

Immunofluorescence analysis was conducted following established protocols as described in previous studies [53]. Mouse brains were fixed in 4% paraformaldehyde at 4 °C for 24 h, followed by dehydration in 20% sucrose solution in PBS at 4 °C overnight, followed by further dehydration in 30% sucrose solution in PBS. Brains were then embedded in OCT medium in an embedding mold and frozen at 0 °C. Embedded tissues were sectioned at 35 μm using a Leica CM3050S cryostat. Brain slices were permeabilized in PBS containing 0.3% Triton X-100 and 5% BSA and then incubated with primary antibody at 4 °C overnight. After three PBS washes, sections were incubated with secondary antibody for 2 h at room temperature. Antifade Mounting Medium with DAPI (Beyotime. P0131) was used to protect sections. The samples were then mounted with Vectashield mounting medium (Vector Laboratories), and images were captured using a Leica TCS SP8 confocal microscope. Primary antibodies used included anti-6E10 (1:500, BioLegend, #SIG-39320), anti-MAP2 (1:500, Merck Millipore, AB5622), anti-GRIN1 (1:200, HUABIO, ET1703-75), anti-neurogranin (1:500, R&D Systems, MAB7947) and anti-Parvalbumin (1:750, SIGMA, P3088). Secondary antibodies used included anti-p Fluor 488-conjugated (goat anti-mouse, A-11029, 1:500, Thermo Fisher) and anti-p Fluor 594-conjugated (goat anti-rabbit, A-11012, 1:500, Thermo Fisher).

#### Acetylated RNA Immunoprecipitation (acRIP)

To quantify ac4C modification levels of specific genes, acetylated RNA immunoprecipitation (acRIP) was performed following a protocol similar to the m6A RNA immunoprecipitation (MeRIP) assay, with the anti-m6A antibody replaced by an anti-ac4C antibody according to the manufacturer’s instructions (Millipore, USA). Briefly, the anti-ac4C antibody (Abcam) was incubated with magnetic beads overnight to allow binding. The antibody-bead complex was then incubated with RNA samples. After RNA was eluted from the beads, RT-qPCR was performed to assess ac4C levels.

#### Quantitative reverse transcription polymerase chain reaction (RT-qPCR)

cDNA was synthesized by reverse transcription using the HiScript III RT SuperMix for qPCR Kit (Vazyme, R323-01). Amplification was then performed using the ChamQ Universal SYBR qPCR Master Mix Kit (Vazyme, Q711-02). Gene expression levels were normalized to Gapdh as an internal control. The primer sequences used for qPCR in this study were as follows:

Grin1: forward 5’-ctgtctcctacacagctggc-3’; reverse 5’-ttctctgccttggactcacg-3’.

Map2: forward 5’-cgtaaatggggatttggtca-3’; reverse 5’-tcgactttccatcccacttc-3’.

Dnajc6: forward 5’-caggcaggctccaagtctac-3’; reverse 5’-cagtccaattcctggtcgct-3’.

Gapdh: forward 5’-gggtgtgaaccacgagaaat-3’; reverse 5’-actgtggtcatgagcccttc-3’.

### Proteomics

#### Sample preparation for proteomic analysis

For each group, hippocampal tissue was collected, washed in PBS, and subjected to global protein extraction using 8 M urea (pH 8.0) with phenylmethanesulfonyl fluoride (PMSF) as a protease inhibitor. The samples were ground using a cryogenic grinder and subjected to sonication for a period of three minutes, with a cycle time of three seconds on and three seconds off, at an amplitude of 25%. The protein concentration was determined using the Bradford assay, and 100 µg of protein was subjected to overnight digestion following the filter-assisted sample preparation (FASP) method with 3.5 µg of trypsin in 50 mM ammonium bicarbonate (pH 8.0) at 37 °C. The resulting peptides were purified by extraction with 50% acetonitrile (ACN) and 0.1% formic acid (FA), desalted using Empore 3 M C18 discs (2 mg, 3 μm, 150 Å, Agela) in pipette tips, and dried using a vacuum concentrator (Thermo Fisher Scientific, USA).

#### Liquid chromatography-tandem mass spectrometry (LC-MS/MS) analysis of the peptide mixture

Proteomic analysis was performed on a nanoElute-HPLC system (Bruker Daltonics) coupled to a hybrid trapped ion mobility spectrometry quadrupole time-of-flight mass spectrometer (TIMS-TOF Pro, Bruker Daltonics, Billerica, MA) using a Captive Spray nano-electrospray ion source. The peptide mixture was reconstituted in solution A (0.1% FA) and loaded onto an analytical column (75 μm i.d. × 25 cm). Peptides were separated over a 60-minute gradient at a flow rate of 600 nL/min: 2–22% solvent B (ACN with 0.1% FA) for 45 min, 22–37% B for 5 min, 37–80% B for 5 min, followed by 80% B for 5 min. Mass spectrometry (MS) analysis was performed in positive electrospray ionization mode over a 100–1700 m/z mass range. Accumulation and ramp times were set to 100 ms each. Full scan MS spectra (m/z 100–1700) were acquired with ion mobility scanning from 0.7 to 1.3 Vs/cm². Each acquisition cycle (1.16 s) consisted of one TIMS MS full scan and 10 parallel accumulation serial fragmentation (PASEF) MS/MS scans. During PASEF, the collision energy was ramped from 59 eV at 1/K0 = 1.6 Vs/cm² to 20 eV at 1/K0 = 0.6 Vs/cm².

#### Proteome identification and quantification using PEAKS-based database searching

Raw MS files were analyzed using PEAKS Online Xpro software (v1.4) for peptide and protein identification by searching the Swiss-Prot database (downloaded on August 20, 2020, containing 20,375 protein entries) and quantification with a match between run (MBR) algorithm. Trypsin was selected as the proteolytic enzyme, allowing for up to three missed cleavage sites. The mass tolerance was set to 15 ppm for precursor ions and 0.05 Da for fragment ions. Oxidation of methionine and N-terminal acetylation were specified as variable modifications. False discovery rates (FDR) for peptide spectrum matches (PSMs) and proteins were kept below 1%. For proteome quantification, peptide feature area values were converted to a fraction of total (FOT) values, then multiplied by 10^6 for ease of presentation. For proteins detected in both of the 5×FAD and WT groups, differential expression analysis was performed using the limma package in R. A protein was considered differentially expressed if the adjusted p-value (adj. p-value) was less than 0.05 and the fold change was greater than or equal to 2. For proteins detected exclusively in either the 5×FAD or WT group, we assumed that these proteins were differentially expressed.

#### acRIP -seq

A total of 10 to 50 micrograms of RNA was subjected to immunoprecipitation using the GenSeq acRIP Kit (GenSeq Inc.) in accordance with the manufacturer’s instructions. In summary, total RNA was randomly fragmented to a length of approximately 200 nt using RNA fragmentation reagents. The anti-ac4C antibody or control IgG (sc-2027, Santa Cruz) was conjugated to Protein A/G Dynabeads (Share-Bio, SB-PR001) in PBS and incubated for one hour at room temperature. Subsequently, the RNA fragments were incubated with the bead-conjugated antibodies, which were rotated at 4 °C for four hours. Subsequently, the RNA/antibody complexes were subjected to multiple rounds of washing to remove any unbound materials. The captured RNA was subsequently eluted from the complexes and purified to ensure its suitability for subsequent analysis. Ribosomal RNA (rRNA) depletion was performed on the RNA samples using the NEBNext rRNA Depletion Kit (New England Biolabs, Inc.). RNA libraries for immunoprecipitation (IP) and input samples were constructed using the NEBNext Ultra II Directional RNA Library Prep Kit (New England Biolabs, Inc.) in accordance with the standard protocol. The quality of the libraries was evaluated using the Agilent 2100 Bioanalyzer (Agilent), and sequencing was conducted on the NovaSeq platform (Illumina).

### Quantification and statistical analysis of the ac4C epitranscriptome

#### Identification of ac4C peaks and positional analysis of acetylated sites

After RNA integrity assessment, acRIP enrichment control and library quality control, raw reads were quality controlled using Q30 and FastQC. Adapter trimming of 3’ sequences and removal of low-quality reads were performed using Cutadapt software (v1.9.3) to generate clean reads. A post-alignment filter was applied to exclude alignments to mitochondrial DNA (chrM) and mismatched mating pairs. Clean reads were then aligned to the mouse genome (mm10) using Hisat2 software (v2.04).

To identify acetylated sites, we evaluated the enrichment of clean reads in acRIP, input and IgG control samples. MACS software (v1.4.2) was used to detect regions of significant read enrichment in the acRIP samples, with adjustments made to optimize the analysis of transcript-mapped reads (e.g., disabling the shift model and local lambda, and setting the sequencing read length to 150 bp). Input samples were used as controls during peak calling. The enriched regions were identified as acetylated sites and ac4C peaks were generated in bed or bam format for further analysis. These peaks were visualized on the UCSC Genome Browser (https://genome.ucsc.edu/cgi-bin/hgGateway) using IGV (http://www.igv.org/).

Strict filtering criteria were applied during ac4C peak calling to ensure data reliability. First, only peaks located on protein-coding exons were selected for further analysis. Additional filters including an adjusted p-value threshold of < 0.05 and a fold enrichment (FE) greater than 1, were used. Peaks with a false discovery rate (FDR)-adjusted p-value < 0.05 were considered statistically significant. A calibration step was performed for IgG control peaks to exclude non-specific IgG binding peaks, retaining only those with no coordinate overlap. Fold enrichment (FE) was calculated by dividing the difference between the acRIP and IgG peaks by the input peak. This procedure ensured that the acetylated regions identified reflected significant and specific ac4C modification levels.

$$\:FE=\frac{acRIP-IgGRIP}{Input}$$

#### Analysis of ac4C fold enrichment (FE) for individual mRNAs

Upon analyzing the fold enrichment (FE) value of each ac4C peak and its corresponding gene, it was observed that multiple ac4C peaks could occur on the same mRNA, rather than a simple one-to-one correspondence between a peak and an mRNA. Consequently, for mRNAs with multiple ac4C-modified regions, the FE of the mRNA was defined as the aggregate of the FE values of the aforementioned peaks. In the case of mRNAs with a single ac4C peak, the FE value of the peak was employed directly in defining the mRNA’s FE value. The following quantification index was used to calculate the ac4C levels in individual mRNAs:

$$\:FE\:\:of\:\:ac4C\:mRNA=\sum\:\:ac4C\:peak(1+2+3\cdots\:+n)$$

#### The e ac4C fold change (FC) in individual mRNAs was analyzed

The FC of ac4C-mRNA reflects the degree of change in the overall acetylation level of mRNA in the WT group compared to the 5×FAD group. To circumvent the potential issue of a zero denominator in the FC calculation, we incorporated a value 1 into the fold enrichment (FE) of ac4C. For ac4C-modified mRNA detected in both the 5×FAD and WT groups, differential expression analysis was performed using the limma package in R. An ac4C-modified mRNA was considered differentially expressed if the adjusted p-value (adj.p.val) was less than 0.05 and the fold change was greater than or equal to 2. For ac4C-modified mRNA detected exclusively in either the 5×FAD or WT group was considered differentially expressed if the fold change was greater than or equal to 2, regardless of statistical significance. The FC of ac4C-mRNA was calculated using the following quantification index:

$$\:FC\:\:of\:\:ac4C\:mRNA=\frac{\left(FE\:\:of\:\:ac4C\:mRNA+1\right)in\:\:5\times\:FAD}{\left(FE\:\:of\:\:ac4C\:mRNA+1\right)in\:\:WT}$$

#### RNA-seq

In summary, total RNA was initially subjected to ribosomal RNA (rRNA) depletion through the use of the GenSeq^®^ rRNA Removal Kit (GenSeq, Inc.), per the manufacturer’s instructions. Subsequently, the rRNA-depleted samples were processed for library construction using the GenSeq^®^ Low Input RNA Library Prep Kit (GenSeq, Inc.). The quality and quantity of the library were evaluated using the Agilent Bioanalyzer 2100 system (Agilent Technologies, Inc., USA). The sequencing was conducted on an Illumina NovaSeq platform, generating of 150 bp paired-end reads.

The quality of the sequencing data was evaluated using Q30 metrics. Low-quality reads and 3’ adapter sequences were removed using the Cutadapt software (version 1.9.3). The remaining high-quality reads were then aligned to the reference genome using the HISAT2 software (v2.0.4). The raw counts were generated using HTSeq software (version 0.9.1). Transcripts per kilobase million (TPM) were calculated to assess similarities and differences between samples. The identification of differentially expressed genes (DEGs) was conducted using the edgeR (v3.16.5) software, which involved normalizing raw counts and calculating fold change between the two sample groups. A gene was considered to be differentially expressed if the p-value was less than 0.05 and the fold change was greater than or equal to 2.

#### Principal component analysis (PCA)

The dimensionality of the omics data was reduced using principal component analysis (PCA) to facilitate the assessment of similarities and differences between samples [54, 55]. For instance, in the context of proteomics data, proteins exhibiting a variance of 1 or less across samples were excluded, as they were deemed inconsequential for PCA analysis. Each protein’s median absolute deviation (MAD) was calculated and subsequently ranked in descending order. Only the top 50% of proteins, based on their MAD values, were selected for PCA to minimize the influence of background noise.

#### Gene ontology and functional category analysis

Gene Ontology (GO) analysis was performed to identify enriched GO terms among differentially acetylated genes and differentially expressed proteins using the clusterProfiler package in R. The functional roles of these genes were categorized into three major GO domains: Molecular Function (MF), Biological Process (BP), and Cellular Component (CC). GO terms with a false discovery rate (FDR)-adjusted p-value of < 0.05 were considered statistically significant and visualized using custom R scripts. In addition, the clusterProfiler package was used to annotate genes involved in the Kyoto Encyclopedia of Genes and Genomes (KEGG) pathways. A p-value threshold of < 0.05 was used to determine significant pathway enrichment, with results visualized accordingly.

#### Protein-protein interaction (PPI) network analysis

Genes consistently downregulated in proteomic and acRIP-seq analyses of 5×FAD mice, relative to WT controls, were selected for further Gene Ontology (GO) analysis. The top five enriched Biological Processes (BP) were then intersected with genes identified as downregulated in human hippocampal proteomics [33] to enhance translational relevance.

To investigate the functional and physical interactions among these genes, a protein-protein interaction (PPI) network was constructed using the STRING database (version 12.0, https://string-db.org/), applying a minimum confidence score of 0.4 to ensure reliable interactions [56]. The PPI network was visualized in Cytoscape (version 3.10.1), and network clusters were further analyzed using the Molecular Complex Detection (MCODE) algorithm (v2.0.3) to identify densely connected components, highlighting potential core modules within the network.

### Statistical analysis

Cumulative distribution was analyzed using the Kolmogorov-Smirnov test in R, while the percentage of ac4C summits within mRNA regions was assessed by chi-square test in R. The CIBERSORT algorithm in R was utilized to identify the enriched cell types associated with the decreased expression of key protein-related genes in the hippocampus of 5×FAD mice. Unless otherwise specified, all other data are presented as mean ± SEM. Sample sizes were determined based on prior studies and met statistical power requirements. Statistical analysis was conducted using an unpaired, two-tailed Student’s t-test for data with a single experimental variable and normal distribution. Two-way ANOVA was applied for datasets with multiple experimental variables, followed by post hoc multiple comparison tests (Tukey, Dunnett, or Sidak) as appropriate. Correlations between bivariate variables were evaluated using the Phi correlation coefficient, with|phi| < 0.3 considered indicative of low relevance. All statistical analyses were performed using GraphPad Prism (version 9.5). Statistical significance is denoted as follows: ****P < 0.0001, ***P < 0.001, **P < 0.01, and *P < 0.05.

The following software and algorithms were utilized for the quantification and statistical analysis of proteomics and ac4C epitranscriptomic data.

**Table:** 

Software and algorithms, application, authors		
R v4.4.0	Statistical computing and graphics	R Core Team, R: A Language and Environment for Statistical Computing, 2024.
ggplot2	Creating graphics, based on The Grammar of Graphics.	Hadley W et al., Springer Verlag New York 2016.
limma	Differential expression analysis of Protein or ac4C mRNA.	Matthew E Ritchie et al., Nucleic Acids Res, 2015.
ComplexHeat map	Arrange multiple heatmaps and support annotation graphics.	Gu Z et al. Bioinformatics 2016.
corrplot	Visualization of a Correlation Matrix	Taiyun Wei and Viliam Simko, 2024.
enrichplot	Visualization of Functional Enrichment Result	Yu G. 2024
circlize	Visualize the location of the target gene in the genome	Gu, Z. 2014
bedtools	Compare large sets of genomic features.	Aaron Q et al. Bioinformatics 2010.
PEAKS Online Xpro Software (v1.4)	peptide and protein identification.	Bioinformatics Solutions Inc., Waterloo, Ontario, Canada
cutadapt v1.9.3	Remove sequencing data adaptor.	Martin M. EMBnet, 2011.
Hisat2 v2.04	Mapping sequencing reads to a single reference genome.	Zhang Y et al. Genome Research 2021.
MACS v1.4.2	Identifying transcript factor binding sites.	Yong Z et al. Genome Biology 2008.
Pandas v2.1.4	Cleaning data.	The pandas development team. Zenodo 2020.
HTSeq (v0.9.1)	Analyzing high-throughput sequencing data.	Simon A et al. Bioinformatics 2014.
edgeR (v3.16.5)	Empirical analysis of digital gene expression data.	McCarthy DJ et al. Nucleic Acids Research 2012.
CIBERSORT	Cell-type identification by estimating relative subsets of RNA transcripts.	Aaron M et al. Nature methods 2015.

## Electronic supplementary material

Below is the link to the electronic supplementary material.

Supplementary Material 1

Supplementary Material 2

Supplementary Material 3

## Data Availability

All data are available in the main text, supplementary or source data files. The original data of acRIP-seq and RNA-seq are accessible via GEO database (GSE281343, GSE281676, GSE285655, and GSE286035). The mass spectrometry proteomics data were deposited at the ProteomeXchange Consortium via the iProX partner repository [57] under the data set identifier PXD057641.

## Abbreviations

ac4C: N4-acetylcytidine
mRNAs: Messenger RNAs
AD: Alzheimer’s disease
WT: Wild-type
acRIP-seq: Acetylated RNA immunoprecipitation sequencing
RNA-seq: RNA sequencing
acRIP: Acetylated RNA immunoprecipitation
RT-Qpcr: Quantitative Reverse Transcription Polymerase Chain Reaction
GO: Gene ontology
BP: Biological Processes
CC: Cellular Components
MF: Molecular Functions
KEGG: Kyoto encyclopedia of genes and genomes
Aβ: Amyloid-beta
m6A: N6-methyladenosine
m5C: 5-methylcytosine
NAT10: N-acetyltransferase 10
PS1: Presenilin-1
FOT: Fraction of Total
MAD: Mean absolute deviation
PCA: Principal component analysis
MBR: Match between run
TPM: Transcripts Per Million
FE: Fold enrichment
FC: Fold change
CDS: Coding DNA sequence
GRIN1: Glutamate ionotropic receptor NMDA type subunit 1
MAP2: Microtubule-associated protein 2
DNAJC6: DnaJ heat shock protein family (Hsp40) member C6
PV: Parvalbumin
NG: Neurogranin
So: Stratum oriens
Sp: Stratum pyramidale
Sr: Stratum radiatum
