# Supplementary figures and images for: Dysregulated ac4C modification of mRNA in a mouse model of early-stage Alzheimer’s disease
Source: Cell Biosci. 2025 Apr 13;15:45. doi: 10.1186/s13578-025-01389-8 (PMC11995559; doi:10.1186/s13578-025-01389-8)

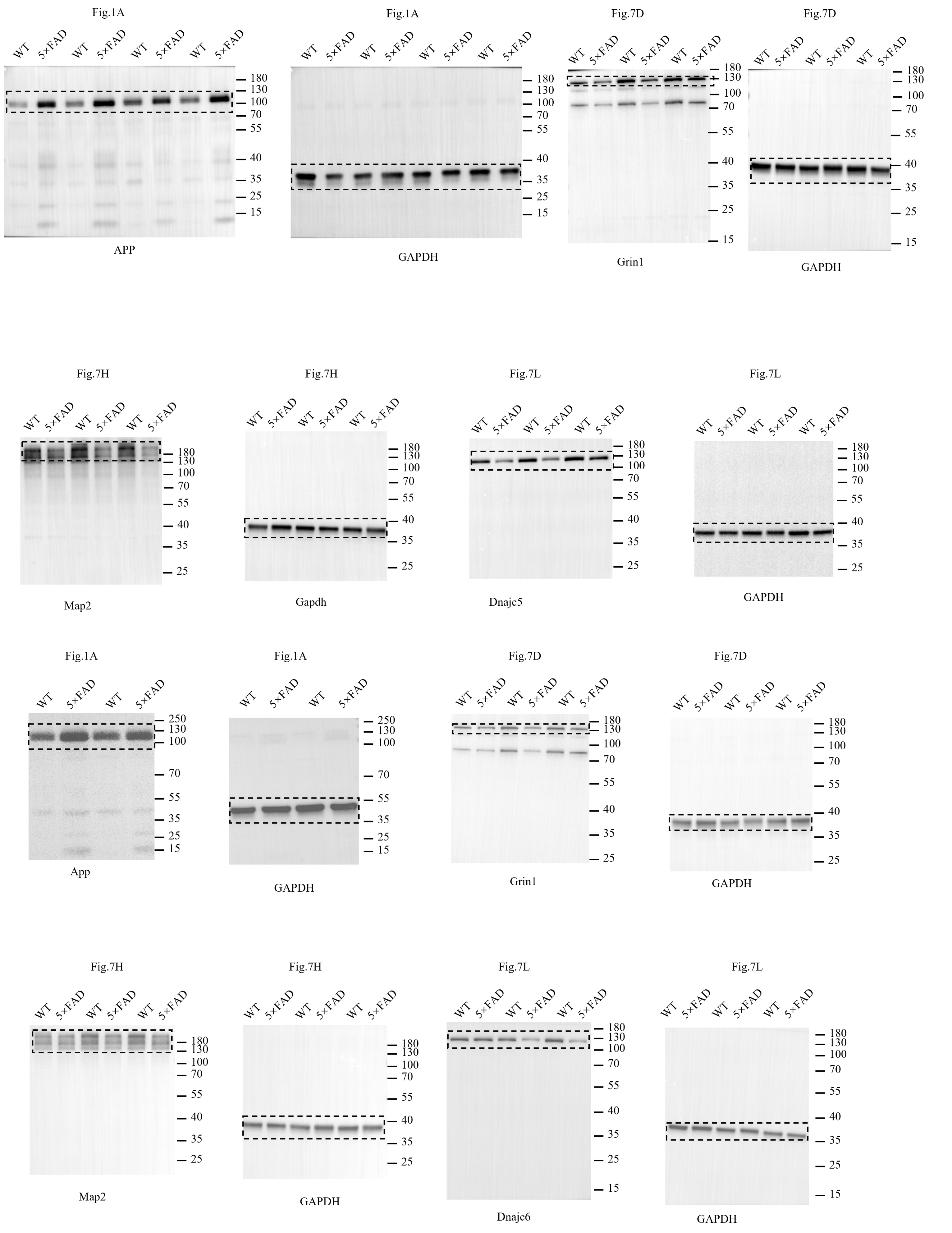

Supplement: Supplementary file 1 — Supplementary Material 1 [file 13578_2025_1389_MOESM1_ESM.tif]

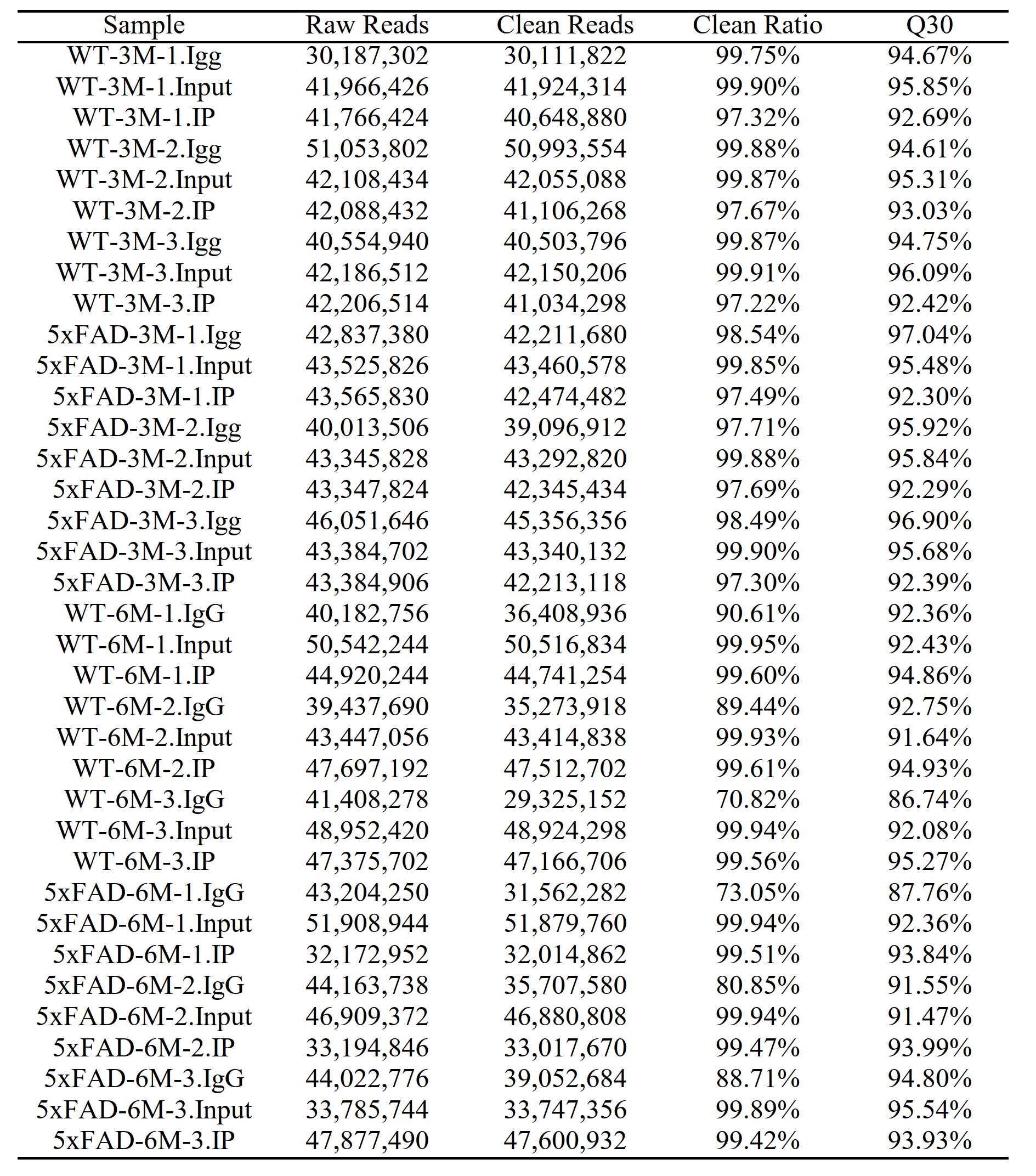

Supplement: Supplementary file 3 — Supplementary Material 3 [file 13578_2025_1389_MOESM3_ESM.tif]
